# Productivity costs from a dengue episode in Asia: a systematic literature review

**DOI:** 10.1186/s12879-020-05109-0

**Published:** 2020-06-03

**Authors:** Trinh Manh Hung, Donald S. Shepard, Alison A. Bettis, Huyen Anh Nguyen, Angela McBride, Hannah E. Clapham, Hugo C. Turner

**Affiliations:** 1 Oxford University Clinical Research Unit, Wellcome Trust Major Overseas Programme, Ho Chi Minh City, Vietnam; 2 Schneider Institutes for Health Policy, Heller School, Brandeis University, Waltham, USA; 3 London Centre for Neglected Tropical Disease Research, London, UK; 4 Department of Infectious Disease Epidemiology, School of Public Health, Faculty of Medicine, St Marys Campus, Imperial College London, Norfolk Place, London, UK; 5 Department of Global Health and Infection, Brighton and Sussex Medical School, Falmer, Brighton, England; 6 Centre for Tropical Medicine and Global Health, Nuffield Department of Medicine, University of Oxford, Oxford, UK; 7 Saw Swee Hock School of Public Health, National University of Singapore, Singapore, Singapore; 8 MRC Centre for Global Infectious Disease Analysis, Department of Infectious Disease Epidemiology, School of Public Health, Faculty of Medicine, St Mary’s Campus, Imperial College London, Norfolk Place, London, UK

**Keywords:** Dengue, Indirect costs, Productivity losses, Productivity costs, Economic burden, Informal caregiver, Asia

## Abstract

**Background:**

Dengue is a mosquito-borne viral infection which has been estimated to cause a global economic burden of US$8.9 billion per year. 40% of this estimate was due to what are known as productivity costs (the costs associated with productivity loss from both paid and unpaid work that results from illness, treatment or premature death). Although productivity costs account for a significant proportion of the estimated economic burden of dengue, the methods used to calculate them are often very variable within health economic studies. The aim of this review was to systematically examine the current estimates of the productivity costs associated with dengue episodes in Asia and to increase awareness surrounding how productivity costs are estimated.

**Method:**

We searched PubMed and Web of Knowledge without date and language restrictions using terms related to dengue and cost and economics burden. The titles and abstracts of publications related to Asia were screened to identify relevant studies. The reported productivity losses and costs of non-fatal and fatal dengue episodes were then described and compared. Costs were adjusted for inflation to 2017 prices.

**Results:**

We reviewed 33 relevant articles, of which 20 studies reported the productivity losses, and 31 studies reported productivity costs. The productivity costs varied between US$6.7–1445.9 and US$3.8–1332 for hospitalized and outpatient non-fatal episodes, respectively. The productivity cost associated with fatal dengue episodes varied between US$12,035-1,453,237. A large degree of this variation was due to the range of different countries being investigated and their corresponding economic status. However, estimates for a given country still showed notable variation.

**Conclusion:**

We found that the estimated productivity costs associated with dengue episodes in Asia are notable. However, owing to the significant variation in methodology and approaches applied, the reported productivity costs of dengue episodes were often not directly comparable across studies. More consistent and transparent methodology regarding the estimation of productivity costs would help the estimates of the economic burden of dengue be more accurate and comparable across studies.

## Background

**Fig. 1:**
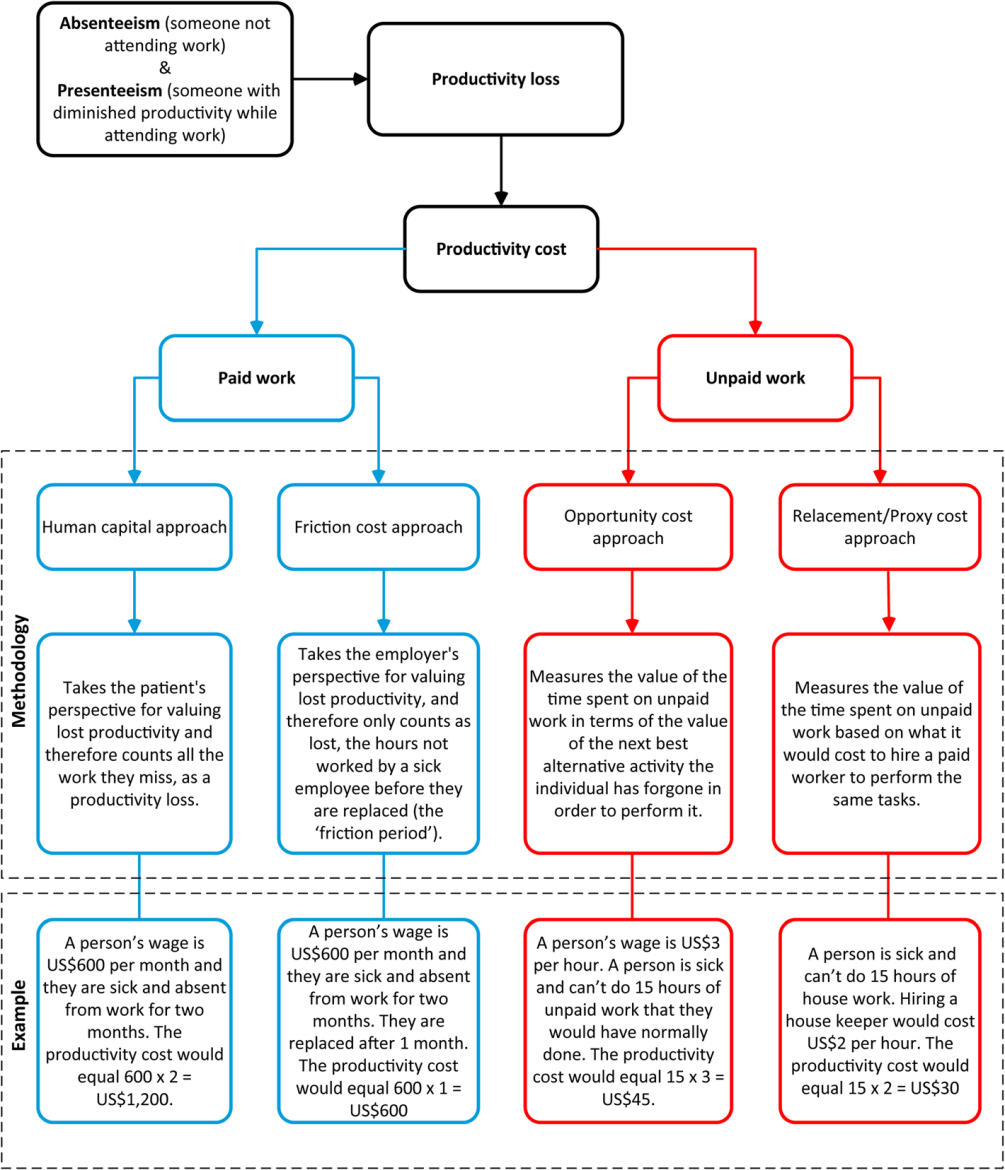
An overview of the methods to value productivity costs

Dengue is a widespread mosquito-borne viral disease, with over 40% (2.5 billion people) of the world’s population at risk of infection [1]. The incidence of dengue has increased 30-fold over the past 50 years, and between 2005 and 2015 the number of dengue-related deaths increased by nearly 50% (from 12,300 to 18,400 deaths annually) [1, 2]. Recently, it was estimated that there were 58.4 million symptomatic dengue cases and 13,586 dengue-related deaths in 2013 [3, 4]. These numbers corresponded to an estimated total cost of illness of US$8.9 billion (2013 prices) [4]. Of this total estimated cost of illness, US$3.77 billion (42%) resulted from productivity costs [5]. Productivity costs (also known as indirect costs) are defined as the costs associated with the loss of paid and unpaid work that result from illness, treatment, disability or premature death [6] i.e. they are the monetary value of productivity losses (lost productive time). These can occur from the patients themselves as well as their informal caregivers. However, although productivity costs accounted for a significant proportion of the estimated economic burden of dengue, the methods used to calculate them are inconsistent across studies (Fig. 1 and Additional file 1). This is not unique to dengue; it is a general problem across the health economics research field [7]. The accurate estimation of productivity costs is important to understand the true economic burden of dengue.

The aim of this review is to systematically examine the current estimates of the productivity costs associated with dengue episodes in Asia, and to gain a more comprehensive understanding regarding the variation in the methodology used to calculate these costs. We also discuss more generally many of the areas of debate surrounding the quantification of productivity costs, how productivity costs can be applied within cost-effectiveness analyses, and make recommendations for future research. These findings will help improve future research and consistency regarding the quantification and reporting of dengue-related productivity costs. This will be helpful for developing more accurate estimates of the economic burden of dengue as well as helping policymakers and healthcare funders in decision making regarding dengue interventions.

## Methods

**Fig. 2:**
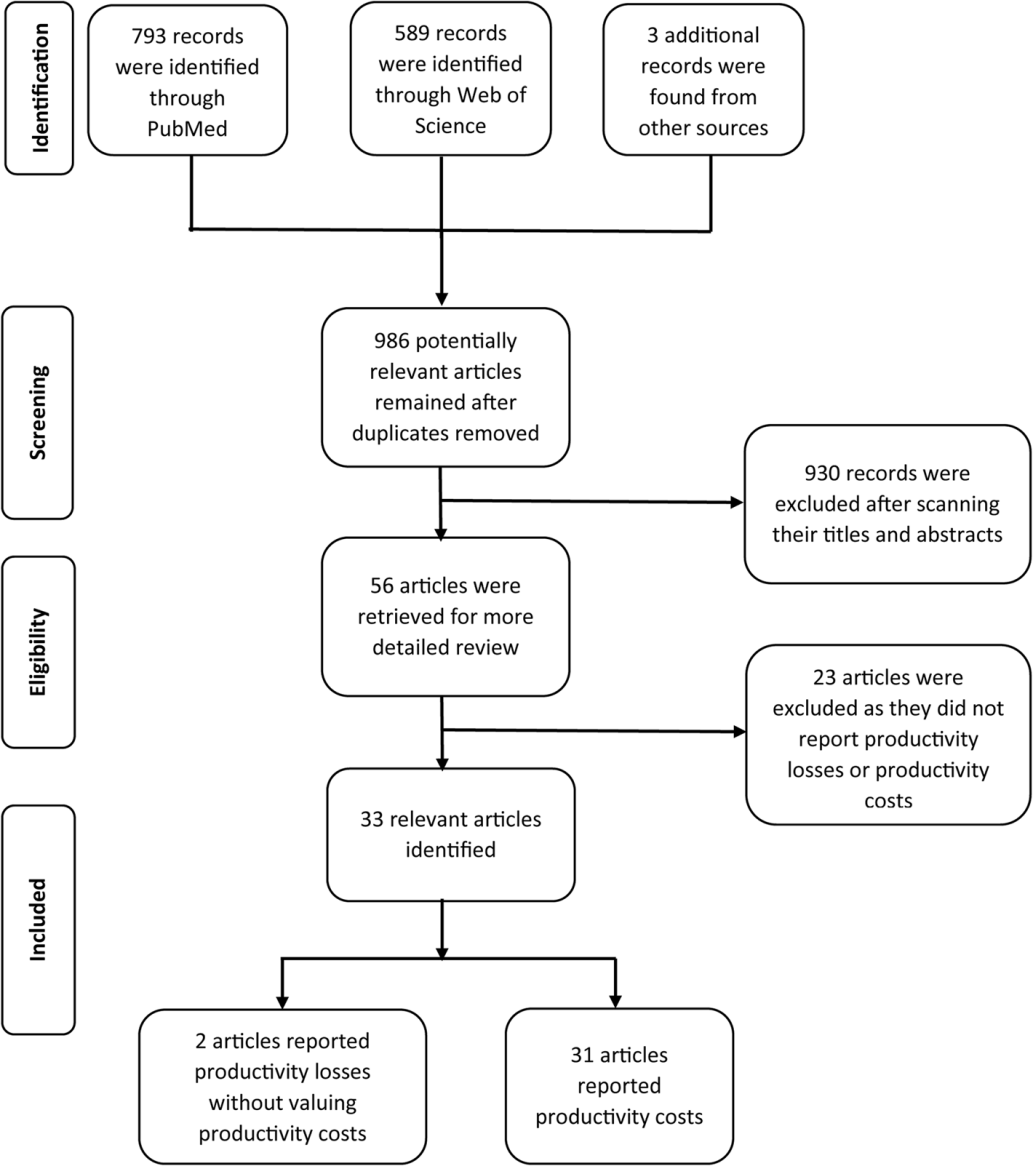
Decision tree outlining the inclusion and exclusion of the identified studies. *Several ‘grey literature’ texts which were not found within the databases were also identified using Google Scholar and the bibliographies of other papers. A PRISMA checklist is provided in* Additional file 2

We searched for relevant articles using the PubMed and Web of Science electronic databases using variants of the following keywords (dengue, dengue virus, dengue hemorrhagic fever, dengue fever, dengue infection, DENV, DENV-1, DENV-2, DENV-3, DENV-4, economics, cost, cost analysis, cost of illness, health care cost, and cost-effectiveness) on 24th of April 2019, without any date or language restrictions. The search terms were discussed and tested by two reviewers (TMH and HCT). A detail information of search terms and PRISMA checklist were provided in Additional file 2. The titles and abstracts of all the articles were scanned to identify relevant studies by a single reviewer (TMH). The bibliographies of related papers were also searched to find additional articles not originally retrieved from the databases. The studies related to the Asia region were screened for manually. Full texts of the identified studies were then reviewed for eligibility by a reviewer (TMH) and articles without reported productivity losses or costs were excluded. Any studies with uncertainty regarding their inclusion were discussed and resolved by two reviewers (TMH and HCT). The full selection process is outlined in Fig. 2.

One key feature of dengue is the variety of types and definitions of dengue episodes. Many studies focus implicitly on episodes managed in the formal health care system, while episodes managed outside the formal healthcare system were commonly excluded [4]. In some non-health economic studies, “cases” also include non-symptomatic dengue infections. Accordingly, in this review, we refer only to symptomatic dengue episodes. Estimates related to the productivity losses and costs of both non-fatal and fatal dengue episodes were extracted. Where feasible, we also reported the estimated productivity losses and costs associated with the patients’ informal caregivers.

When possible, we stratified the results according to the severity of the dengue episode (such as dengue fever, severe or non-severe dengue, dengue hemorrhagic fever and dengue shock syndrome) and the source of care (outpatient versus hospitalized). Notably, the World Health Organization (WHO) classification for dengue has been updated from dengue fever or dengue hemorrhagic fever to dengue or severe dengue [8]. We reported the estimates in the same way as in the source paper.

Throughout this paper, productivity losses were defined as the estimated amount of productive time (i.e. paid or unpaid work) lost due to a dengue episode. Productivity costs were defined as the monetary value of these productivity losses.

As the studies were performed in different years, it was necessary to adjust for inflation to make the estimates more directly comparable. We therefore adjusted the reported productivity costs to 2017 US prices. For the majority of the studies, the reported productivity costs were converted to their country’s currency and adjusted for inflation using the specific country’s Gross Domestic Product (GDP) deflator rates from the International Monetary Fund and then reconverted to US$ using the 2017 exchange rate [9–11]. Further detail is provided in the Additional file 2.

## Results

We identified 56 potentially relevant articles reporting the cost of dengue illness in Asian countries. Of these, 23 of the studies only reported direct costs and were therefore excluded. The remaining 33 studies were included in the analysis and reviewed in further detail. Details of the identified articles are described below (Tables 1 and 2). Generally, the majority of the studies (83%) focused on countries in Southeast Asia that are considered to be highly endemic. Moreover, the majority of the studies (64%) were published within the last 10 years. A notable gap in the literature was the relatively few studies performed in India and China.

**Table 1:** The estimated productivity costs of non-fatal dengue episodes

Authors	Countries	Wage source	Caregiver’s productivity costs included	Average productivity cost related to a non-fatal dengue episode (US$ 2017 prices)
**Hospitalized episodes**				
Clark et al. [12]	Thailand	Unclear	Yes	37.5
Huy et al. [13]	Cambodia	From survey	Yes	14.1
Mia et al. [14]	Malaysia	Minimum wage	Yes	276.5
Okanurak et al. [15]	Thailand	Minimum wage of two different cities	Yes	Adult patient: 54.3 Child patient: 64.5
Suaya et al. [16]	Cambodia	Minimum wage	Yes	85.9
	Thailand	Minimum wage	Yes	67.9
	Malaysia	Minimum wage	Yes	143.0
Kongsin et al. [17]	Thailand	Minimum wage	Yes	68.4
Suaya et al. [18]	Cambodia	Minimum wage	Yes	Average: 85.2 Plasma leakage case: 91.4 Non-plasma leakage case: 65.1
Onuh et al. [19]	Philippines	Average minimum wage for non-agriculture and agriculture sectors	NA	57.7
Tam et al. [20]	Vietnam	From survey	NA	64.7
Pham et al. [21]	Vietnam	From survey	NA	51.3
Nguyen et al. [22]	Vietnam	From survey	Yes	Adult patient: DF: 13.5 DHF: 15.1 DSS: 16.8 Child patient: DF: 9.7 DHF: 18.1 DSS: 26.3
Suaya et al. [23]	Cambodia	Unclear	Yes	30.4
Harving et al. [24]	Vietnam	From survey	Yes	24.2
Shepard et al. [25]	Malaysia	Estimation	NA	196.0
Lee et al. [26]	Vietnam	From survey	Yes	58.0
	Thailand	From survey	Yes	46.4
Bhavsar et al. [27]	India	From survey and minimum wage	Yes	155.1
Tran et al. [28]	Vietnam	GDP per capita and from survey	Yes	75.7
Tozan et al. [29]	Thailand	Minimum wage		Adult patient: Average: 101.2 DF: 87.9 DHF: 134.7 Child patient: Average: 84.1 DF: 84.8 DHF: 80.6
Tran et al. [30]	Vietnam	From survey	Yes	Median: 88.1
Fezzazi et al. [31]	Indonesia	Daily wage	Caregiver only	Overall average: 231.8
	Malaysia			
	Philippines			
	Thailand			
	Vietnam			
Zeng et al. [32]	Indonesia	GDP per capita	NA	60.4
	Malaysia	GDP per capita	NA	181.7
	Philippines	GDP per capita	NA	41.9
	Thailand	GDP per capita	NA	50.9
	Vietnam	GDP per capita	NA	15.4
Shepard et al. [33]	Bhutan	GDP per capita	NA	37.3
	Brunei	GDP per capita	NA	615.8
	Cambodia	GDP per capita	NA	39.1
	East Timor	GDP per capita	NA	9.6
	Indonesia	GDP per capita	NA	48.0
	Laos	GDP per capita	NA	20.5
	Malaysia	GDP per capita	NA	198.6
	Myanmar^a^	GDP per capita	NA	11.9
	Philippines	GDP per capita	NA	42.5
	Singapore	GDP per capita	NA	1445.9
	Thailand	GDP per capita	NA	54.6
	Vietnam	GDP per capita	NA	10.7
Shepard et al. [4]	Afghanistan	GDP per capita	NA	6.7
	Bangladesh	GDP per capita	NA	13.7
	Bhutan	GDP per capita	NA	39.7
	Brunei	GDP per capita	NA	927.7
	Cambodia	GDP per capita	NA	38.1
	China	GDP per capita	NA	127.3
	India	GDP per capita	NA	19.5
	Indonesia	GDP per capita	NA	52.3
	Lao	GDP per capita	NA	22.8
	Malaysia	GDP per capita	NA	168
	Maldives	GDP per capita	NA	141.3
	Myanmar	GDP per capita	NA	12.1
	Nepal	GDP per capita	NA	8.1
	Pakistan	GDP per capita	NA	19
	Philippines	GDP per capita	NA	38.2
	Singapore	GDP per capita	NA	911.6
	Srilanka	GDP per capita	NA	51.2
	Taiwan	GDP per capita	NA	1318.4
	Tajikistan	GDP per capita	NA	7.6
	Thailand	GDP per capita	NA	50.7
	East Timor	GDP per capita	NA	74.9
	Uzbekistan	GDP per capita	NA	10.2
	Vietnam	GDP per capita	NA	13.2
	Yemen	GDP per capita	NA	31.6
Nadjib et al. [34]	Indonesia	From survey and minimum wage	Yes	45.0–130.2
Lee et al. [35]	Cambodia	From survey	Yes	59.9
Bangert et al. [36]	Maldives	Unclear	NA	134.4
**Outpatient episodes**				
Shepard et al. [25]	Malaysia	Minimum wage	NA	172.5
Fezzazi et al. [31] ^a^	Indonesia Malaysia Philippines Thailand Vietnam	Average wage	Caregiver only	Overall average: 65.8
Lee et al. [26]	Vietnam	From survey	Yes	23.4
	Thailand	From survey	Yes	27.7
Suaya et al. [16]	Cambodia	Minimum wage	NA	NA
	Thailand	Minimum wage	Yes	125.2
	Malaysia	Minimum wage	NA	NA
Zeng et al. [32]	Indonesia	GDP per capita	NA	33.4
	Malaysia	GDP per capita	NA	159.6
	Philippines	GDP per capita	NA	22.8
	Thailand	GDP per capita	NA	12.5
	Vietnam	GDP per capita	NA	11.3
Tran et al. [30]	Vietnam	From survey	Yes	Median: 44.1
Shepard et al. [33]	Bhutan	GDP per capita	NA	17.5
	Brunei	GDP per capita	NA	288.7
	Cambodia	GDP per capita	NA	5.6
	East Timor	GDP per capita	NA	4.5
	Indonesia	GDP per capita	NA	22.5
	Laos	GDP per capita	NA	9.6
	Malaysia	GDP per capita	NA	173.8
	Myanmar^a^	GDP per capita	NA	5.6
	Philippines	GDP per capita	NA	19.9
	Singapore	GDP per capita	NA	1332.1
	Thailand	GDP per capita	NA	13.7
	Vietnam	GDP per capita	NA	8.4
Shepard et al. [4]	Afghanistan	GDP per capita	NA	3.8
	Bangladesh	GDP per capita	NA	7.5
	Bhutan	GDP per capita	NA	21.5
	Brunei	GDP per capita	NA	519.7
	Cambodia	GDP per capita	NA	5.6
	China	GDP per capita	NA	71.8
	India	GDP per capita	NA	11.3
	Indonesia	GDP per capita	NA	29.4
	Lao	GDP per capita	NA	12.5
	Malaysia	GDP per capita	NA	146.8
	Maldives	GDP per capita	NA	79.4
	Myanmar	GDP per capita	NA	6.9
	Nepal	GDP per capita	NA	4.6
	Pakistan	GDP per capita	NA	10.7
	Philippines	GDP per capita	NA	21.8
	Singapore	GDP per capita	NA	839.4
	Srilanka	GDP per capita	NA	28.5
	Taiwan	GDP per capita	NA	739
	Tajikistan	GDP per capita	NA	4.4
	Thailand	GDP per capita	NA	12.4
	East Timor	GDP per capita	NA	41.8
	Uzbekistan	GDP per capita	NA	5.5
	Vietnam	GDP per capita	NA	10.2
	Yemen	GDP per capita	NA	18.3
Nadjib et al. [34]	Indonesia	From survey and minimum wage	Yes	8.1–24.6
Bangert et al. [36]	Maldives	Unclear	NA	75.5
**Unspecified setting**				
Luh et al. [41]^α^	Taiwan	GDP per capita	NA	The average annual productivity cost per case Epidemic year: 176.4 Non-epidemic year: 169.3
Carrasco et al. [37]	Singapore	GDP per capita	NA	HCA: 1669.8–3198.9 FCA: 1247.3–2281.7
Hariharan et al. [38]	India	GNI per capita	NA	12.7

Where possible the costs were adjusted to US$ 2017 prices (see Additional file 2). In some cases, the adjusted costs were smaller than the original values reported due to changes in the country’s US$ exchange rate

*IS* International dollars, *NA* not available, *GDP* gross domestic product, *HCA* human capital approach, *FCA* friction cost approach, *GNI* gross national income, *DF* dengue fever, *DHF* dengue hemorrhagic fever, *DSS* dengue shock syndrome

^a^: US GDP deflators were used to adjust for inflation (see Additional file 2)

α: Values were not adjusted for inflation (see Additional file 2)

**Table 2:** The estimated average productivity costs related to fatal dengue episodes

Authors	Country	Method	Wage source	Discount rate	Average productivity cost related to a fatal dengue episode (US$ 2017 prices)
Okanurak et al. [15]	Thailand	NA	GNP per capita	NA	Children: 166,861 Adult: 167,209
Suaya et al. [16]	Cambodia	NA	GDP per capita	3%	15,568
	Thailand	NA	GDP per capita		99,339
	Malaysia	NA	GDP per capita		132,074
Shim et al. [39]	Philippines	HCA	GDP per capita	3%	Children: 85,218 Adults: 55,392
Carrasco et al. [37]	Singapore	HCA and FCA	HCA used GDP per capita, FCA used gross earning for friction period	3%	HCA: 396,836.8-417,187.4 FCA: 5698.2-7122.7
Shepard et al. [25]	Malaysia	HCA	Minimum wage	3%	52,168.9
Beaute et al. [40]	Cambodia	HCA	Average annual income from World Bank	3%	16,242.1
Luh et al.^α^ [41]	Taiwan	HCA	GDP per capita	3%	Epidemic year: 224,884.4 Non-epidemic year: 215,634.7
Shepard et al. [4]	Afghanistan	HCA	GDP per capita	3%	Children: 18,077 Adult: 12,368
	Bangladesh	HCA	GDP per capita	3%	Children: 34,772 Adult: 22,354
	Bhutan	HCA	GDP per capita	3%	Children: 77,077 Adult: 49,873
	Brunei	HCA	GDP per capita	3%	Children: 851,115 Adult: 554,098
	Cambodia	HCA	GDP per capita	3%	Children: 32,483 Adult: 21,282
	China	HCA	GDP per capita	3%	Children: 187,606 Adult: 122,518
	India	HCA	GDP per capita	3%	Children: 44,120 Adult: 28,729
	Indonesia	HCA	GDP per capita	3%	Children: 91,835 Adult: 59,693
	Lao	HCA	GDP per capita	3%	Children: 49,823 Adult: 32,178
	Malaysia	HCA	GDP per capita	3%	Children: 238,657 Adult: 155,441
	Maldives	HCA	GDP per capita	3%	Children: 208,713 Adult: 135,881
	Myanmar	HCA	GDP per capita	3%	Children: 28,443 Adult: 18,962
	Nepal	HCA	GDP per capita	3%	Children: 23,007 Adult: 14,955
	Pakistan	HCA	GDP per capita	3%	Children: 43,947 Adult: 28,506
	Philippines	HCA	GDP per capita	3%	Children: 72,754 Adult: 47,290
	Singapore	HCA	GDP per capita	3%	Children: 1,453,237 Adult: 948,121
	Sri Lanka	HCA	GDP per capita	3%	Children: 93,487 Adult: 61,013
	Taiwan	HCA	GDP per capita	3%	Children: 1,199,795 Adult: 783,068
	Tajikistan	HCA	GDP per capita	3%	Children: 19,002 Adult: 12,035
	Thailand	HCA	GDP per capita	3%	Children: 159,870 Adult: 104,347
	East Timor	HCA	GDP per capita	3%	Children: 121,101 Adult: 79,282
	Uzbekistan	HCA	GDP per capita	3%	Children: 21,256 Adult: 13,777
	Vietnam	HCA	GDP per capita	3%	Children: 55,848 Adult: 36,555
	Yemen	HCA	GDP per capita	3%	Children: 69,819 Adult: 46,546
Hariharan et al. [38]	India	NA	NA	NA	33,185.4
Bangert et al. [36]	Maldives	NA	NA	NA	Children: 198,181.3 Adult: 129,024.3

Where possible, the costs were adjusted to US$ 2017 prices. In some cases, the adjusted costs were smaller than the original values reported due to changes in the country’s US$ exchange rate

α: Values were not adjusted for inflation (see Additional file 2)

Human capital and friction cost approaches are defined in Fig. 1

*GNP* Gross national product, *GDP* gross domestic product, *HCA* human capital approach, *FCA* friction cost approach, *NA* not available

There was substantial variation regarding how the different studies defined and estimated productivity losses and productivity costs. For instance, some studies only considered the productivity losses of the patients, while others also accounted for the losses experienced by the patients’ informal caregivers (Additional file 2 Table S1). There was also notable variation in how the studies placed monetary values on productivity losses, i.e. how they estimated the productivity costs. Many studies did not collect primary data and based their estimates on secondary or aggregated data, particularly for the productivity costs relating to premature mortality.

In the following sections we summarize:

1. The reported productivity losses (i.e. the productive time lost) associated with non-fatal dengue episodes and their informal caregivers.
2. The estimated productivity costs associated with non-fatal episodes i.e. the monetary value of the productivity losses.
3. The estimated productivity costs related to fatal dengue episodes.
4. How these estimated productivity costs of dengue episodes influence estimates of dengue’s total economic burden.
5. How these productivity costs can be incorporated into cost-effectiveness analyses of dengue interventions.

### The reported productivity losses related to non-fatal dengue episodes

#### Patients’ productivity losses (productive time lost)

A number of studies reported the productivity losses associated with dengue episodes (Additional file 2 Table S1). The majority of the studies collected this data via interviews with patients and/or their caregivers. The average reported number of days loss from a dengue episode occurring in a child ranged between 3.9–11.4 days for hospitalized episodes and 0.7–4.3 days for outpatient episodes. Similarly, the reported number of days loss from a dengue episode occurring in an adult ranged from 5 to 31.9 days for hospitalized episodes and 4.0–7.2 for outpatient episodes. However, there were differences in the way the different studies estimated and reported productivity losses, particularly in children (Additional file 2 Table S1), which made a formal comparison of the different studies difficult. For instance, to estimate the productivity losses for children, the studies varied with some using the number of school days they had missed, the duration of the illness [35, 42], the number of work days loss by their informal caregivers [22, 34, 41] or a combination of factors [15, 17, 21]. However, the duration of illness does not necessarily equal the number of school days loss (such as if a child is ill over a weekend). In addition, the time that informal caregivers spent looking after sick children can be substantial [13, 18], and thus this also needs to be accounted for to fully capture the productivity losses of dengue episodes occurring in children.

All of the studies quantified productivity losses relating to absenteeism (someone not attending work). However, none of the studies appeared to explicitly quantify the productivity losses relating to presenteeism. Presenteeism is likely to be present at every phase of the illness. It is probable that presenteeism is even more significant in low and lower-middle-income countries, as the patients will be less likely to have access to paid “sick days” and many would not be able to afford to take much time off work. Studies on arthritis and mental health have found that presenteeism accounted for the majority of their associated total productivity losses [43–45]. Compared to long-term chronic illnesses, the impact of presenteeism will likely be less extreme for dengue, however it should be considered and evaluated further in future studies.

The patients’ productivity losses were found to correspond to the severity of the dengue episode. Three studies differentiated the productive time lost based on disease severity [17, 22, 41]. For example, Nguyen et al. [22] found that adult patients lost on average 8.4, 9.7 and 12.3 work days with dengue fever, dengue hemorrhagic fever, and dengue shock syndrome cases, respectively. Lee et al. [26] also found that the number of sick days reported before and after study enrollment were significantly related to the severity of illness.

The majority of studies focused implicitly on episodes managed within the formal health system. Although this is understandable, this focus excludes episodes that cause productivity losses but do not receive a formal diagnosis or management in the formal health care system.

**Fig. 3:**
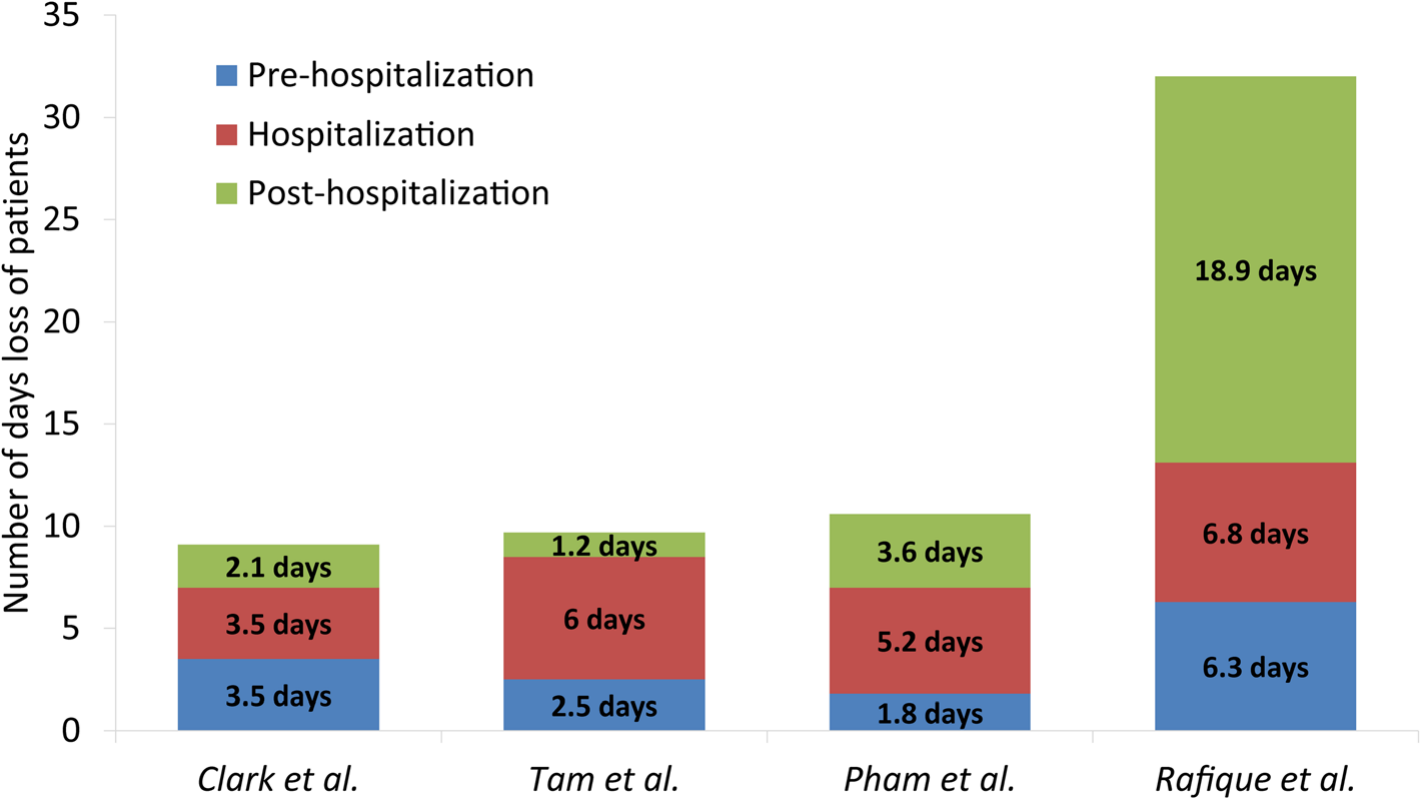
The average number of days lost by patients over the whole course of a dengue episode. *Clark* et al. [12], *Pham* et al. [21], *Rafique* et al. [46], *Tam* et al. [20]*. It should be noted only these papers were included as they were the only ones that reported the days lost stratified in this way*

Four studies reported the patients’ productivity losses pre, during and post-hospitalization period [12, 20, 21, 46]. They ranged from 1.8–6.25 days for pre-hospitalization period, 3.5–6.76 days for hospitalization period and 1.2–18.89 days for post-hospitalization period (Fig. 3). This demonstrates the importance of also quantifying the productivity losses experienced pre- and post-hospitalization. It is noteworthy that the productivity losses estimated by Rafique et al. [46] were much larger than the other studies, particularly those experienced post-hospitalization. The authors stated that this difference could be potentially due to patients experiencing fatigue post-infection [46, 47].

Within the literature, there is variation regarding the reported proportion of cases (2–68%) experiencing post-acute consequences of dengue infection (such as fatigue, asthenia and trouble working), as well as the reported duration of these symptoms [48]. The majority of the studies we identified within this review would not have a long enough follow-up period to fully capture the potential impact of these post-acute consequences on the estimated productivity losses of dengue. The incidence, duration and severity of these post-acute consequences is a key area of uncertainty regarding the burden of dengue [49]. Currently, the majority of studies in this area are from Latin America, and more research is needed from other settings (particularly higher transmission settings, where the age of distribution of episodes differs) [49].

#### Unpaid informal caregivers’ productivity losses (productive time lost)

Many dengue patients require support from informal caregivers: individuals (often family members) who provide unpaid assistance and support while the patient is ill. Within the studies we identified, the average number of days lost by the patients’ informal caregivers ranged between 1.0–25.5 days for hospitalized episodes and 0.1–2 days for outpatient episodes (Additional file 2 Table S1). The number of days informal caregivers spent looking after patients did not necessarily equal their number of work days loss. For instance, Huy et al. [13] reported that informal caregivers spent on average 11.4 days looking after a dengue patient, whilst on average, they lost 8.3 work days. Only capturing the number of work days loss might underestimate the actual productivity losses of informal caregivers. For example, some informal caregivers may not be working (such as students or retirees). This highlights the importance of also considering lost unpaid work when estimating productivity losses.

As with the estimated productivity losses of the patients, the productivity losses of the informal caregivers corresponded to the severity of the episode [18, 22]. For instance, Suaya et al. [18] reported that families of children with plasma leakage (a marker of more severe dengue) lost on average five days more than those without plasma leakage (15.2 versus 10.5 days). Similarly, Nguyen and Luong [22] reported that informal caregivers lost an average of 7.3, 7.1 and 9.9 days for dengue fever, dengue hemorrhagic fever and dengue shock syndrome cases, respectively.

In some instances, multiple caregivers will support a single dengue patient. However, only a few studies reported the actual number of informal caregivers that were supporting the dengue patients [12, 22, 41, 46]. If this is not accounted for, the productivity losses of informal caregivers could be underestimated. Future studies should therefore clearly state the number of informal caregivers and their assumptions when calculating these productivity losses.

It was not always clear if studies were quantifying the productivity losses experienced by informal caregivers across the full duration of the dengue episodes or just period that the patient is hospitalized/at the outpatient clinic. If the former was done, the total productivity losses experienced by dengue patients’ informal caregivers could have been underestimated in some studies.

### The estimated productivity costs related to non-fatal dengue episodes

**Fig. 4:**
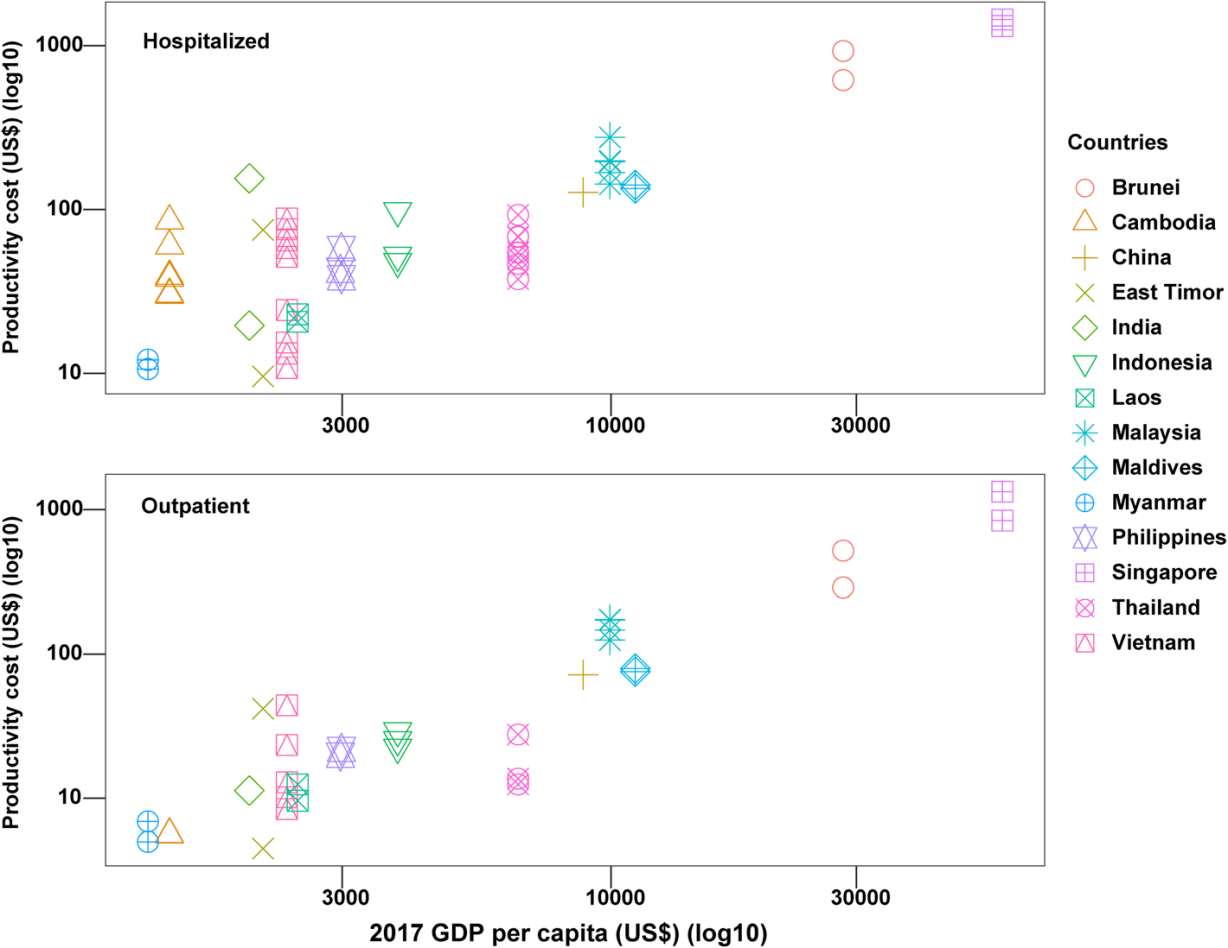
The reported average productivity costs in the identified studies. *Costs are reported in 2017 prices*

In order to estimate the productivity costs associated with dengue episodes, a monetary value is placed on the corresponding productivity losses (i.e. number of days loss) discussed previously. The estimated productivity costs from the different studies are summarized in Table 1. However, owing to the variety and inconsistency in the methods applied to calculate the productivity costs, it was difficult to directly compare the estimates. Accordingly, the estimated productivity costs associated with dengue episodes varied widely across the different studies. Specifically, the average estimated productivity costs of a hospitalized episode ranged from US$6.7–1445.9 (2017 prices) and the average estimated productivity cost of an outpatient episode ranged from US$3.8–1332 (2017 prices) (Table 1). A large degree of this variation was due to the range of different countries being investigated and their corresponding economic status. Unsurprisingly, there was a positive relationship between the estimated productivity cost and the country’s GDP (Fig. 4). However, estimates for a given country still showed notable variation (Additional file 2 Figure S1). This was likely largely due to differences in methodology, such as the wage sources used to value productivity losses and whether or not the productivity losses of informal caregivers were included.

The methods for placing a monetary value on productivity losses can vary depending on whether the loss is experienced by an adult patient, a child patient or an informal caregiver. These issues are discussed more in the following subsections.

#### Valuing monetarily an adult’s productivity losses (estimating productivity costs)

**Fig. 5:**
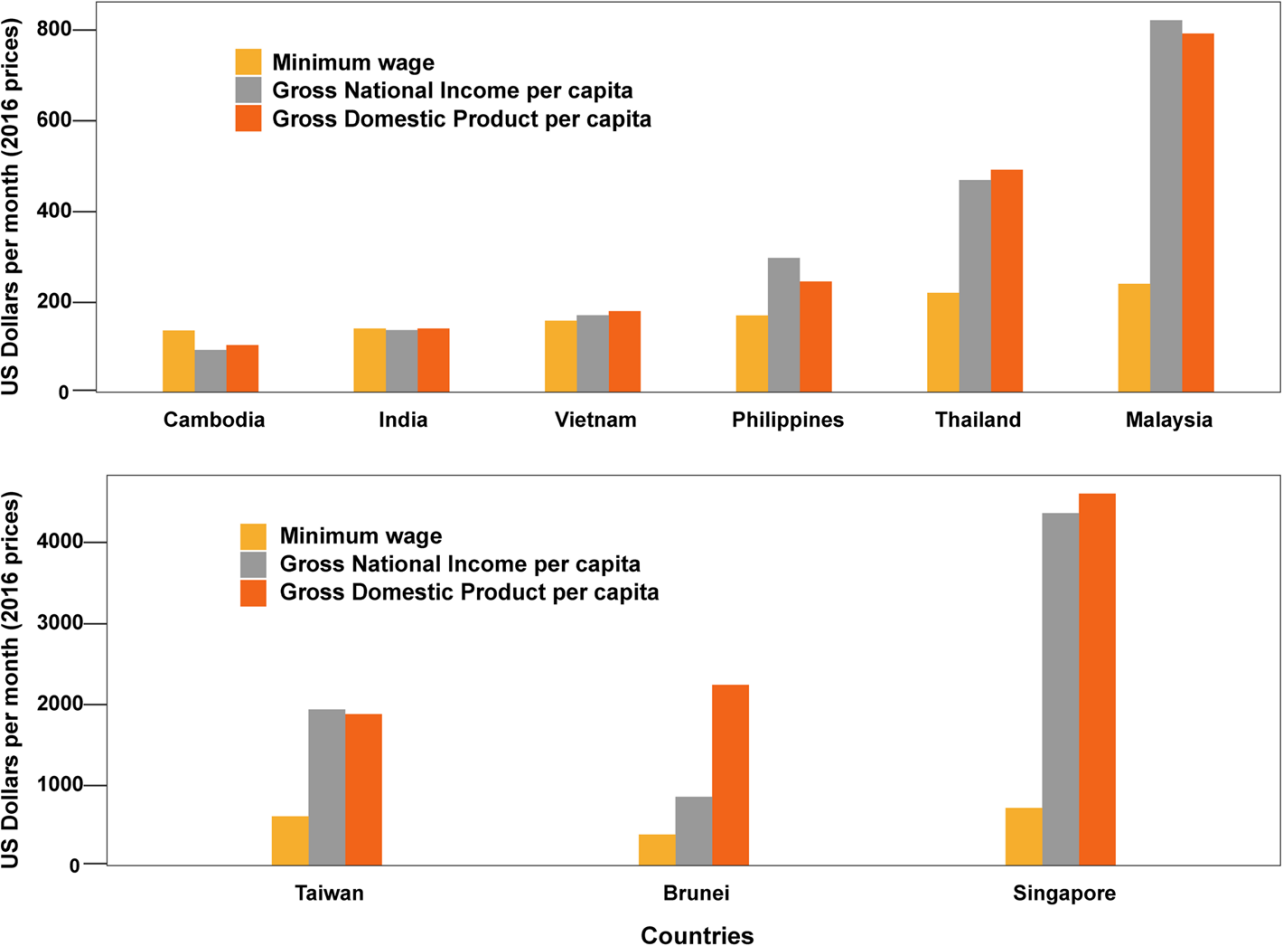
The difference in some example potential wage sources commonly used to value productivity losses. *Data sources: Minimum wage* [50–58], *Gross National Income* [59], *and Gross Domestic Product* [60]*. Costs are reported in 2016 US$ prices*

The productivity losses incurred by adult dengue patients are typically valued based on a specified wage source(s), which reflects the average income of the population at risk of disease in the study setting. Many of the identified studies used a minimum wage for this [14–19, 25, 27]. However, the minimum wage does not always reflect the actual average income of the whole population (Fig. 5). Furthermore, there is currently no definitive evidence that dengue is strongly associated with poverty [61, 62]. Accordingly, using the minimum wage for valuing monetarily dengue-related productivity losses could lead to an underestimation of the corresponding productivity costs. The extent of this will vary depending on the study setting (Fig. 5).

Currently, there is no standard method for estimating productivity costs. This has resulted in variation across different studies, even when they are reporting to have used the same approach; this is true not just for dengue but for the health economics field in general [7]. For example, some countries have different types of minimum wage (such as Vietnam, where it varies across different regions and the Philippines where it varies for different types of occupation). However, studies often do not clearly state specifically what minimum wage they are using and its source. In addition, variations in how wage sources are converted/modified for the purposes of the study can have a significant impact (e.g., to estimate a daily wage is the yearly wage rate divided by 365 days or by the number of work days etc.).

A key area of debate in the health economic field is regarding the use of the human capital approach or the friction cost approach (Fig. 1 and Additional file 1) and the two approaches can lead to significantly different estimates of productivity costs [63]. However, for non-fatal dengue episodes, it could be argued that there would often be little difference between the two approaches, as the typical duration of illness is shorter than the friction period. Interestingly, despite the approach not always being stated directly, most of the studies appeared to have used/applied the human capital approach to estimate the productivity costs related to non-fatal and fatal dengue episodes.

The productivity costs of lost paid work were commonly quantified and reported across the studies. However, those related to lost unpaid work were often ignored. Specifically, some studies did not value the productivity losses of unemployed patients, students or individuals not earning money at the time of the survey (such as farmers). It should be acknowledged that quantifying monetarily unpaid productivity losses is challenging, and the correct methodology is debatable. Though there are some recommendations [64], there are no specific guidelines on how to measure and value unpaid productivity losses within cost of illness studies. More research and development of guidelines are urgently needed to inform practice in this area [65].

#### Valuing monetarily children’s productivity losses

Valuing monetarily the productivity losses of children is challenging, and currently there is no broadly accepted guideline or methodology for this [64, 66]. Because of this, there was notable variation regarding how the productivity costs relating to dengue episodes in children were estimated. For example, Kongsin et al. [17] estimated this productivity cost by multiplying the cost that the government spends per day on a primary school student and the number of school days loss, while other researchers such as Okanurak et al. [15] and Nadjib et al. [34] based the estimate on the productivity losses of the children’s informal caregivers. It is noteworthy that some studies reported the productivity losses of children without quantifying the productivity costs and vice versa [15, 21]. Accordingly, further comparison across studies is limited. Further research and guidelines are needed to allow the productivity costs relating to children to be estimated more accurately and consistently. Some recommendations to help with this area are outlined in Table 3 and discussed further by Andronis et al. [67].

**Table 3:** Research needs and recommendations for future studies

**Key research needs**	
More data quantifying the productivity losses over the full course of the dengue episode.	
More data quantifying the productivity losses associated with presenteeism and lost unpaid work.	
More data quantifying the productivity losses associated with post-acute consequences.	
Further investigation and data describing patents’ informal caregivers and their productivity losses.	
More data on the health and economic burden of dengue from India and China.	
**Recommendations for future studies**	
Clearly state the losses and costs associated with lost paid work and unpaid work.	
Clearly report the period of disease that accounted for the productive time lost (i.e. pre-hospitalization, hospitalization and post-hospitalization).	
Clearly state and justify the wage source(s) used to value the productivity costs.	
Report the average number of informal caregivers per dengue patient and how their time was valued.	
Clearly state and justify how children’s productivity losses are quantified and valued monetarily.	

#### Valuing monetarily informal caregivers’ productivity losses

The time that informal caregivers spend caring for a patient is typically considered to be unpaid work. Therefore, the opportunity cost method or replacement cost method are generally used to quantify the productivity losses of informal caregivers in monetary terms (Fig. 1). The average reported productivity cost of informal caregivers varied between US$7.1–99.4 per hospitalized dengue episode and US$2.1–16.2 per outpatient dengue episode. It should be noted that some studies quantified the productivity cost of informal caregivers but did not report the specific economic loss relating to them (instead, they reported the total productivity cost relating to the dengue episode). Most of the studies appeared to use the opportunity cost approach to value productivity losses of informal caregivers.

The majority of the studies did not specifically report informal caregivers who were teenagers or retirees. Teenagers and retirees could conceivably spend a lot of time caring for patients, though similar to children, quantifying their productivity costs can be challenging and the correct methodology to use is under debate [64, 68, 69].

More transparency is needed in future studies regarding how the productivity costs related to patients’ informal caregivers are quantified.

### The estimated productivity costs related to fatal dengue episodes

We have discussed the estimated productivity costs associated with acute non-fatal dengue episodes; however, a small proportion of dengue patients die. Some studies estimated the economic burden associated with these fatal dengue episodes. The average estimated productivity costs related to a fatal episode varied across the different studies between US$15,568-1,453,237 for episodes in children and US$12,035–948,121 for episodes in adults (Table 2). This variation was partly due to the differences in economic status of the countries investigated, but also due to three main methodological factors/assumptions: the assumed number of potential years of life lost, the wage source and method applied to value the years of life lost. These differences make it difficult to directly compare the estimates from the different studies. To improve consistency, Shepard et al. [4] provided separate estimates for premature deaths in children and adults. Due to the higher number of life years lost by child deaths, their economic values were higher.

The method applied to calculate the number of potential years of productive life lost can influence the estimated productivity costs related to fatal dengue episodes. Different studies used different methods for this [70]. For example, some estimated the potential years of productive life lost based on life expectancy whereas others used retirement age. The majority of studies used the local life expectancy; however, this might overestimate the productivity costs related to premature mortality (as the life expectancy will typically be higher than the retirement age in that setting). Similarly, there was variation in the working ages assumed across studies and countries as productivity losses/costs were quantified from different ages, such as age 15 [29, 40] and age 21 [19]. The appropriate age will depend on the study setting(s) but should be clearly stated and justified.

There was variation across the different studies regarding which wage source was used to value potential years of productive life lost (Table 2). The majority of studies used the per capita GDP [4, 16, 37, 39, 41]. However, the WHO’s cost-effectiveness analysis guide criticized the use of per capita GDP in this way, stating that it would overestimate the productivity costs [71]. Depending on the wage source used, the productivity costs could easily be overestimated or underestimated (Fig. 5). Thus, the wage source employed should be carefully examined such that it appropriately reflects the monetary value of the productivity of the specific population at risk of dengue within the investigated setting(s).

Finally, whether the human capital approach or friction cost approach was used influences the productivity costs related to premature mortality (Fig. 1 and Additional file 1). The estimated productivity cost related to fatal dengue episodes were notably lower when the friction cost approach was used (Table 2). The debate regarding which method is more appropriate is ongoing (Additional file 1). However, even when studies stated that they used the same approach, owing to the variation in the methods outlined above, the results could still be significantly different. Although the friction cost method was proposed as an alternative to the human capital approach [63], the human capital approach was still more commonly used within the field [63].

## Discussion

We found that the estimated productivity costs associated with dengue episodes in Asia are notable. However, due to the significant variation in methodology and approaches applied, the reported productivity costs were often not directly comparable across studies.

In the future, greater consistency regarding how the productivity costs related to dengue are calculated would be invaluable and make different studies more comparable. This will be important for estimates of the total economic burden of dengue and within economic evaluations of dengue interventions.

### The total economic burden associated with productivity costs

We have reviewed the productivity costs associated with individual dengue episodes. It is important to note that the total economic burden associated with dengue-related productivity costs will also depend on the incidence of symptomatic dengue episodes. The Global Burden of Disease (GBD) 2017 study estimated that there were 86,183,620 symptomatic dengue episodes in Asia in 2017 (95% uncertainty interval 51,918,388-131,567,619) [72]. This number is significantly higher than the estimate within the GBD 2013 study (46,059,323 episodes in South Asia and Southeast Asia, East Asia and Oceania) [4]. This highlights the variation in the estimates of the incidence of dengue episodes and the need for this to be considered when comparing the results of different studies. For example, the estimates of the total economic burden of dengue in Vietnam have varied between US$5.43–94.87 million (2016 prices) and this variation was largely driven by the wide range in the assumed incidence of symptomatic episodes (between 69,680-2,263,880 symptomatic dengue episodes per year – based on a range of sources/methods) [73]. This highlights that for a good cost of illness estimates both good cost data and epidemiology data are needed.

### Using productivity costs within cost-effectiveness analysis

Productivity costs account for a significant proportion of the economic burden of dengue [4]. Consequently, if and how they are included within a dengue related cost-effectiveness analysis could notably influence its outcome.

**Fig. 6:**
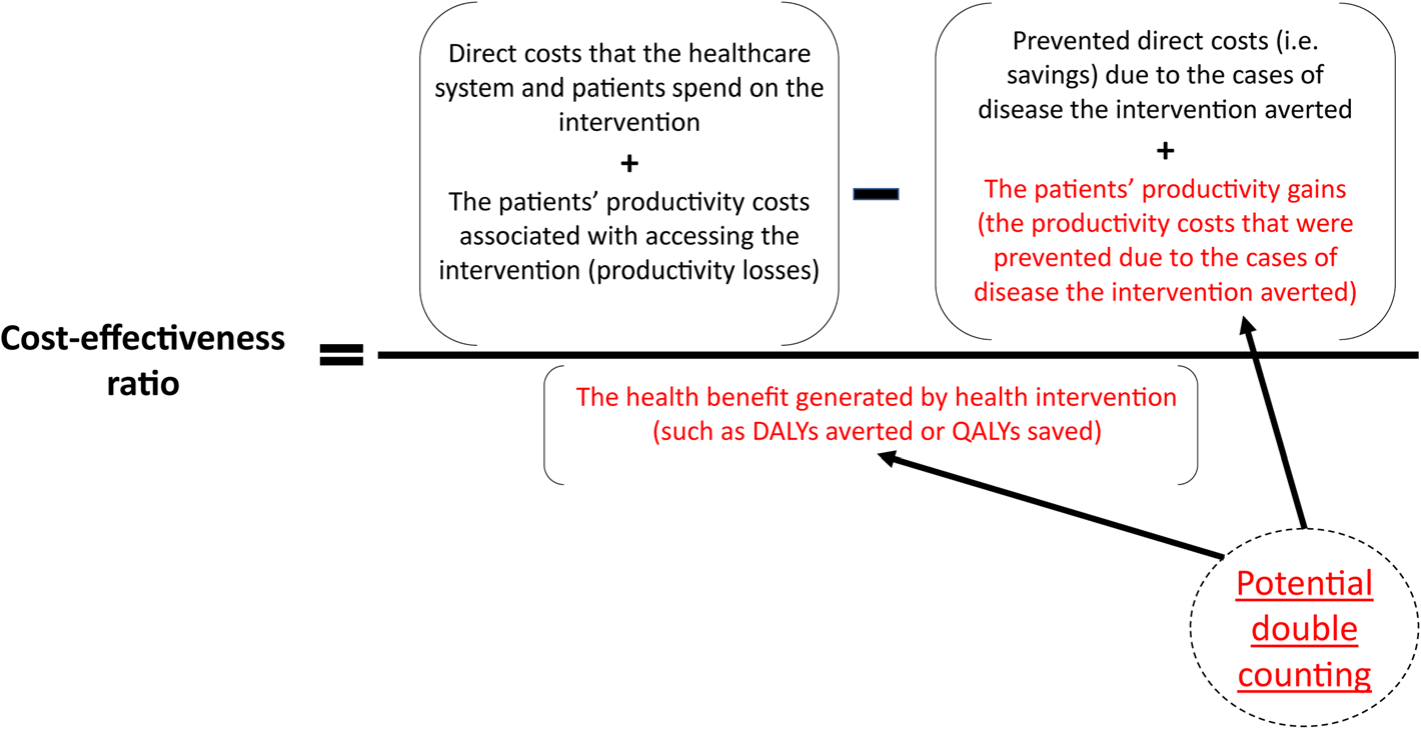
Overview of the types of productivity costs included within cost-effectiveness analysis

Within cost-effectiveness analyses, there are effectively two types of productivity costs (Fig. 6):

- Those corresponding to the productivity losses directly associated with accessing the intervention (e.g. someone takes a day off work to go to an outpatient clinic to get a vaccine).
- Those corresponding to the averted productivity losses that result from prevented morbidity/mortality (i.e. disease case is prevented and therefore, its related productivity costs that would have otherwise occurred are averted). These averted productivity losses are often referred to as productivity gains.

The productivity costs of dengue episodes discussed in the previous sections of this paper could be used to quantify the productivity costs associated with the productivity gains that result from a dengue intervention within a cost-effectiveness analysis (based on the number of episodes averted by an intervention and their corresponding productivity costs). Within the cost-effectiveness ratio, these productivity costs associated with productivity gains are effectively a negative cost (Fig. 6).

The recommendations for which productivity costs should be included in cost-effectiveness analysis vary (Fig. 6). The first United States (US) Panel on Cost-Effectiveness in Health and Medicine (known as the Washington panel) recommended that the productivity costs related to the productivity losses associated with an intervention should be included within the cost component of the cost-effectiveness analysis [74]. However, they argued that those related to the productivity gains associated with prevented morbidity/mortality should not be, as they are (at least partly) captured within the quality-adjusted life year (QALY) effectiveness measure: therefore including these productivity gains within the cost component of the equation would potentially lead to double counting of the effectiveness of the intervention [74]. However, this recommendation has been challenged, with some arguing that the QALY measure does not capture these productivity gains [6, 75–79]. Recently, the second US Panel on Cost-Effectiveness in Health and Medicine recommended that both productivity losses and productivity gains should be included in the cost component of the cost-effectiveness ratio [64]. The debate in this area has heavily focused on the use of QALYs, although it could be argued that there is still a degree of double counting (albeit smaller) with the disability-adjusted life year (DALY) measure.

The WHO’s cost-effectiveness analysis guide recommends that the productivity costs associated with both productivity losses and productivity gains should be excluded from a cost-effectiveness ratio [71]. The reason given was that there is no “conceptually appropriate” way of measuring these productivity changes in monetary terms and that including productivity costs within cost-effectiveness analyses would simply introduce noise into the calculations. They suggested that when productivity gains/losses are believed to be important, studies should attempt to quantify them as rigorously as possible and report them separately.

Owing to the debate in this area within the health economic field, and the significance of the dengue related productivity costs, researchers performing cost-effectiveness analyses of dengue interventions should clearly state their assumptions regarding the methodology being applied and highlight the fact that the appropriateness of certain methods is still under debate.

### Limitations of this analysis

A potential source of bias in our search strategy is that it did not capture studies published outside of the searched electronic databases (i.e. grey literature such as policy documents/reports, and many non-English language publications etc.). For example, we found few articles reporting the burden of dengue in China and India, which may be due to studies relating to these countries not being published in journals which are indexed in PubMed or Web of Science. Efforts were made to minimize this bias by searching Google Scholar, WHO’s Dengue Bulletin and the bibliographies of selected studies. It should be noted that the selection process was not performed independently by two researchers (i.e. not in duplicate), which could result in selection bias. To mitigate this, the papers where there was uncertainty regarding their inclusion were discussed between two reviewers. Furthermore, because there is no standard methodology for conducting cost of illness studies, the quality of the identified studies was not assessed. The variation in quality could result in biases in the estimated productivity costs of dengue episodes (potentially resulting in either an over or underestimation). However, this observed variation further supports our primary finding regarding the need for greater consistency in future studies.

## Conclusion

A major impetus for calculating the cost of an illness episode is to have a standard burden measure that can be used for comparisons across countries and time periods, and for use within economic evaluation of interventions. Productivity costs can be a key component of the cost of an illness dengue episode. We found that the estimated productivity costs associated with dengue episodes in Asia are notable. However, owing to the significant variation in methodology and approaches applied, the reported productivity costs of dengue episodes were often not directly comparable across studies.

The majority of studies we identified only valued absenteeism from paid work. The losses associated with lost unpaid work and presenteeism have not been adequately addressed or characterized. Further work is also needed to account for the productivity costs incurred by patients’ informal caregivers. Due to such losses not being fully captured, the productivity costs of dengue could be underestimated.

In the future, further guidelines and recommendations regarding how to calculate productivity costs would be invaluable and make different studies more consistent and comparable. What is most important moving forward is that the assumptions and methods used to estimate productivity costs are clearly reported.

More consistent and transparent methodology regarding the estimation of productivity costs would help the estimates of the economic burden of dengue be more accurate and comparable across studies. Ultimately, accurate estimates of the economic burden of dengue will be crucial to informing policy in both the prevention and treatment of this highly prevalent disease, particularly in settings with limited resources.

## Supplementary information

**Additional file 1.** Overview of the methods applied to calculate productivity costs.

**Additional file 2 **Description of the adjustment for inflation, **Table S1**: The estimated productivity losses of patients and their informal caregivers, **Figure S1**: The average reported productivity costs as a fraction of the study settings per capita GDP, The search terms and PRISMA CHECKLIST.

## Data Availability

Not applicable.

## Abbreviations

WHO: World Health Organization
GDP: Gross Domestic Product
GNP: Gross National Product
GBD: Global Burden of Disease
US: United States
QALY: Quality-adjusted life year
DALY: Disability-adjusted life year
GNI: Gross National Income
HCA: Human capital approach
FCA: Friction cost approach

## References

[1] World Health Organization (2016). Vector borne diseases.

[2] Murray CJ, Wang H, Naghavi M, Allen C, Barber RM, Bhutta ZA (2016). Global, regional, and national life expectancy, all-cause mortality, and cause-specific mortality for 249 causes of death, 1980–2015: a systematic analysis for the global burden of disease study 2015. Lancet.

[3] Stanaway JD, Shepard DS, Undurraga EA, Halasa YA, Coffeng LE, Brady OJ (2016). The global burden of dengue: an analysis from the global burden of disease study 2013. Lancet Infect Dis.

[4] Shepard DS, Undurraga EA, Halasa YA, Stanaway JD (2016). The global economic burden of dengue: a systematic analysis. Lancet Infect Dis.

[5] Koopmanschap MA, van Ineveld BM (1992). Towards a new approach for estimating indirect costs of disease. Soc Sci Med.

[6] Brouwer WB, Koopmanschap MA, Rutten FF (1997). Productivity costs in cost-effectiveness analysis: numerator or denominator: a further discussion. Health Econ.

[7] Krol M, Brouwer W, Rutten F (2013). Productivity costs in economic evaluations: past, present, future. Pharmacoeconomics..

[8] World Health Organization (2019). Dengue and Severe Dengue.

[9] International Moneytary Fund (2018). Gross Domestic Product, Deflator.

[10] World Bank (2018). Official exchange rate.

[11] Turner HC, Lauer JA, Tran BX, Teerawattananon Y, Jit M. Adjusting for Inflation and Currency Changes Within Health Economic Studies. Value Health. 2019;22(9):1026-32. 10.1016/j.jval.2019.03.021 31511179

[12] Clark DV, Mammen MP, Nisalak A, Puthimethee V, Endy TP (2005). Economic impact of dengue fever/dengue hemorrhagic fever in Thailand at the family and population levels. Am J Trop Med Hyg..

[13] Huy R, Wichmann O, Beatty M, Ngan C, Duong S, Margolis HS (2009). Cost of dengue and other febrile illnesses to households in rural Cambodia: a prospective community-based case-control study. BMC Public Health.

[14] Mia MS, Begum RA, Er AC, Pereira JJ (2016). Assessing the cost burden of dengue infection to households in Seremban, Malaysia. Southeast Asian J Trop Med Public Health..

[15] Okanurak K, Sornmani S, Indaratna K (1997). The cost of dengue hemorrhagic fever in Thailand. Southeast Asian J Trop Med Public Health.

[16] Suaya JA, Shepard DS, Siqueira JB, Martelli CT, Lum LC, Tan LH (2009). Cost of dengue cases in eight countries in the Americas and Asia: a prospective study. Am J Trop Med Hyg..

[17] Kongsin S, Jiamton S, Suaya JA, Vasanawathana S, Sirisuvan P, Shepard DS (2010). Cost of dengue in Thailand. Dengue Bull.

[18] Suaya JA, Chantha N, Huy R, Sah BK, Moh-Seng C, Socheat D (2010). Clinical characterization, diagnosis and socioeconomic impact of hospitalized dengue in Cambodia. Dengue Bull..

[19] Onuh W, Cabanacan-Salibay C, Manaig P (2016). Economic costs and burden of dengue disease in Cavite Province, Philippines. Sci Med.

[20] Tam PT, Dat NT, Huu LM, Thi XCP, Duc HM, Tu TC (2012). High household economic burden caused by hospitalization of patients with severe dengue fever cases in Can Tho province, Vietnam. Am J Trop Med Hyg.

[21] Pham LD, Tran NHP, Le NDT, Vo TQ (2016). Economic report on the cost of dengue fever in Vietnam: case of a provincial hospital. Clinicoecon Outcomes Res.

[22] Nguyen TKT, Luong CQ (2011). Assessing the economic burden of dengue in southern Viet Nam: results of a prospective multicenter cost study.

[23] Suaya JA, Shepard DS, Chang MS, Caram M, Hoyer S, Socheat D (2007). Cost-effectiveness of annual targeted larviciding campaigns in Cambodia against the dengue vector Aedes aegypti. Tropical Med Int Health.

[24] Harving ML, Rönsholt FF (2007). The economic impact of dengue hemorrhagic fever on family level in southern Vietnam. Dan Med Bull.

[25] Shepard DS, Undurraga EA, Lees RS, Halasa Y, Lum LC, Ng CW (2012). Use of multiple data sources to estimate the economic cost of dengue illness in Malaysia. Am J Trop Med Hyg..

[26] Lee JS, Mogasale V, Lim JK, Carabali M, Lee KS, Sirivichayakul C (2017). A multi-country study of the economic burden of dengue fever: Vietnam, Thailand, and Colombia. PLoS Negl Trop Dis.

[27] Bhavsar AT, Shepard DS, Suaya JA, Mafowosofo M, Hurley CL (2010). A private hospital-based study assessing knowledge, attitudes, practices and costs associated with dengue illness in Surat. India Dengue Bull.

[28] Tran NYN, Vo QT (2016). The economic value of informal care for dengue patients in Vietnam. Int J Res Ayurveda Pharm.

[29] Tozan Y, Ratanawong P, Sewe MO, Wilder-Smith A, Kittayapong P (2017). Household costs of hospitalized dengue illness in semi-rural Thailand. PLoS Negl Trop Dis.

[30] Tran BX, Thu GV, Hoang LN, Tuan ALN, Thanh TT, Thanh BN, et al. Cost-of-Illness and the Health-Related Quality of Life of Patients in the Dengue Fever Outbreak in Hanoi in 2017. Int J Environ Res Public Health. 2018;15(6):1174. 10.3390/ijerph15061174 PMC6025163 29874790

[31] El Fezzazi H, Branchu M, Carrasquilla G, Pitisuttithum P, Perroud AP, Frago C (2017). Resource use and costs of dengue: analysis of data from phase III efficacy studies of a tetravalent dengue vaccine. Am J Trop Med Hyg..

[32] Zeng W, Halasa-Rappel YA, Baurin N, Coudeville L, Shepard DS (2018). Cost-effectiveness of dengue vaccination in ten endemic countries. Vaccine..

[33] Shepard DS, Undurraga EA, Halasa YA (2013). Economic and disease burden of dengue in Southeast Asia. PLoS Negl Trop Dis.

[34] Nadjib M, Setiawan E, Putri S, Nealon J, Beucher S, Hadinegoro SR (2019). Economic burden of dengue in Indonesia. PLoS Negl Trop Dis.

[35] Lee J-S, Mogasale V, Lim JK, Ly S, Lee KS, Sorn S (2019). A multi-country study of the economic burden of dengue fever based on patient-specific field surveys in Burkina Faso, Kenya, and Cambodia. PLoS Negl Trop Dis.

[36] Bangert M, Latheef AT, Pant SD, Ahmed IN, Saleem S, Rafeeq FN (2018). Economic analysis of dengue prevention and case management in the Maldives. PLoS Negl Trop Dis.

[37] Carrasco LR, Lee LK, Lee VJ, Ooi EE, Shepard DS, Thein TL (2011). Economic impact of dengue illness and the cost-effectiveness of future vaccination programs in Singapore. PLoS Negl Trop Dis.

[38] Hariharan D, Das MK, Shepard DS, Arora NK. Economic burden of dengue illness in India from 2013 to 2016: A systematic analysis. Int J Infect Dis. 2019;84:S68-73. 10.1016/j.ijid.2019.01.010 30641201

[39] Shim E (2016). Dengue dynamics and vaccine cost-effectiveness analysis in the Philippines. Am J Trop Med Hyg.

[40] Beaute J, Vong S (2010). Cost and disease burden of dengue in Cambodia. BMC Public Health.

[41] Luh DL, Liu CC, Luo YR, Chen SC. Economic cost and burden of dengue during epidemics and non-epidemic years in Taiwan. J Infect Public Health. 2017;11(2):215-23. 10.1016/j.jiph.2017.07.021 28757293

[42] Anderson KB, Chunsuttiwat S, Nisalak A, Mammen MP, Libraty DH, Rothman AL (2007). Burden of symptomatic dengue infection in children at primary school in Thailand: a prospective study. Lancet.

[43] Li X, Gignac MA, Anis AH (2006). The indirect costs of arthritis resulting from unemployment, reduced performance, and occupational changes while at work. Med Care.

[44] Burton WN, Chen C-Y, Conti DJ, Schultz AB, Pransky G, Edington DW (2005). The association of health risks with on-the-job productivity. J Occup Environ Med.

[45] Goetzel RZ, Long SR, Ozminkowski RJ, Hawkins K, Wang S, Lynch W (2004). Health, absence, disability, and presenteeism cost estimates of certain physical and mental health conditions affecting US employers. J Occup Environ Med.

[46] Rafique I, Nadeem Saqib MA, Munir MA, Qureshi H, Siddiqui S, Habibullah S (2015). Economic burden of dengue in four major cities of Pakistan during 2011. J Pak Med Assoc.

[47] Seet RC, Quek AM, Lim EC (2007). Post-infectious fatigue syndrome in dengue infection. J Clin Virol.

[48] Tiga DC, Undurraga EA, Ramos-Castañeda J, Martínez-Vega RA, Tschampl CA, Shepard DS (2016). Persistent symptoms of dengue: estimates of the incremental disease and economic burden in Mexico. Am J Trop Med Hyg..

[49] Hung TM, Wills B, Clapham HE, Yacoub S, Turner HC (2019). The uncertainty surrounding the burden of post-acute consequences of dengue infection. Trends Parasitol.

[50] WageIndicator (2018). Minimum wage in Vietnam with effect from 01–01–2016 to 31-12-2016.

[51] WageIndicator (2018). Minimum Wages in Cambodia with effect from 01–01–2016 to 31-12-2016.

[52] WageIndicator (2018). Minimum wage in Delhi w.e.f April 1, 2016 to September 30, 2016.

[53] Trading economics (2018). Malaysia Minimum Monthly Wages 2013-2018.

[54] Trading economics (2018). Thailand Minimum Daily Wage 1973-2018.

[55] Minimum-wage (2018). Philippines minimum wage rate 2018.

[56] Trading economics (2018). Taiwan Minimum Monthly Wage.

[57] Varkkey B, Korde R, Singh S (2016). *minimum* Wage Comparison: Asian Countries – Minimum Wage Fixing.

[58] The Manila Times (2015). DOLE:Minimum salary of domestic workers in Brunei now is USD 400.

[59] World Bank (2018). GNI per capita, Atlas method (Current US$).

[60] World Bank (2018). GDP per capita (Current US$).

[61] Mulligan K, Dixon J, Sinn C-LJ, Elliott SJ (2015). Is dengue a disease of poverty? A systematic review. Pathog Glob Health.

[62] Teixeira MG, Barreto ML, Costa MCN, Ferreira LDA, Vasconcelos PF, Cairncross S (2002). Dynamics of dengue virus circulation: a silent epidemic in a complex urban area. Tropical Med Int Health.

[63] Koopmanschap MA, Rutten FF, van Ineveld BM, Van Roijen L (1995). The friction cost method for measuring indirect costs of disease. J Health Econ.

[64] Neumann PJ, Sanders GD, Russell LB, Siegel JE, Ganiats TG. Cost-effectiveness in health and medicine, Second edn. New York: Oxford University Press; 2016.

[65] Verbooy K, Hoefman R, van Exel J, Brouwer W (2018). Time is money: investigating the value of leisure time and unpaid work. Value Health.

[66] Castro MC, Wilson ME, Bloom DE (2017). Disease and economic burdens of dengue. Lancet Infect Dis.

[67] Andronis L, Maredza M, Petrou S (2019). Measuring, valuing and including forgone childhood education and leisure time costs in economic evaluation: methods, challenges and the way forward. Soc Sci Med.

[68] Nyman JA (2018). Cost recommendations in the second edition of cost-effectiveness in health and medicine: a review. MDM Policy Pract.

[69] Lakdawalla DN, Doshi JA, Garrison LP, Phelps CE, Basu A, Danzon PM (2018). Defining elements of value in health care—a health economics approach: an ISPOR special task force report [3]. Value Health.

[70] Gardner JW, Sanborn JS (1990). Years of potential life lost (YPLL)—what does it measure?. Epidemiology..

[71] World Health Organization (2003). Making choice in health: WHO guide to cost effective analysis.

[72] IHME (2019). GBD Results Tools.

[73] Hung TM, Clapham HE, Bettis AA, Cuong HQ, Thwaites GE, Wills BA (2018). The estimates of the health and economic burden of dengue in Vietnam. Trends Parasitol.

[74] Gold MR, Siegel JE, Russell LB, Weinstein MC (1996). Cost-effectiveness in health and medicine.

[75] Brouwer WB, Koopmanschap MA, Rutten FF (1997). Productivity costs measurement through quality of life? A response to the recommendation of the Washington panel. Health Econ.

[76] Drummond MF, Sculpher MJ, Claxton K, Stoddart GL, Torrance GW. Methods for the economic evaluation of health care programmes, 4th edn. New York: Oxford University Press; 2015.

[77] Sculpher M. The role and estimation of productivity costs in economic evaluation. In: Economic evaluation in health care: Merging theory with practice. edn. Edited by Michael MD, Alistair McGuire. New York: Oxford University Press; 2001. p. 94-112.

[78] Olsen JA, Richardson J (1999). Production gains from health care: what should be included in cost-effectiveness analyses?. Soc Sci Med.

[79] Liljas B (1998). How to calculate indirect costs in economic evaluations. Pharmacoeconomics..

