# Supplementary material for: Productivity costs from a dengue episode in Asia: a systematic literature review
Source: BMC Infect Dis. 2020 Jun 3;20:393. doi: 10.1186/s12879-020-05109-0 (PMC7268537; doi:10.1186/s12879-020-05109-0)
Supplement: Supplementary file 1 — Additional file 1. Overview of the methods applied to calculate productivity costs. [file 12879_2020_5109_MOESM1_ESM.docx]

**Current methods being applied to calculate productivity costs**

Productivity costs (also known as indirect costs) are defined as the costs associated with the loss of paid and unpaid work that result from illness, treatment, disability or premature death (S1) i.e. they are the monetary value of productivity losses (lost productive time). These can occur from the patients themselves as well as their informal caregivers.

Productivity losses of paid work can result from absenteeism (someone not attending work) and presenteeism (someone’s diminished productivity (in terms of quantity and/or quality) while attending work) (S2). These losses can be valued by using either the human capital approach or the friction cost approach, and there is continued debate regarding which method is most appropriate (S3, S4) (Figure 1). For example, the human capital approach has been criticized for overestimating productivity costs, because it typically assumes that time lost by an individual in the labor market is not compensated for by an otherwise unproductive person (i.e. it does not account for the fact that an ill employee will eventually be replaced) (S5). It could be argued that this assumption does not hold unless unemployment rates are low (S6). To account for this, Koopmanschap *et al.* (S7) proposed the friction cost approach. This takes the employer's perspective for valuing productivity losses and only counts as lost, the hours not worked by a sick employee before they are replaced (S7). However, others have criticized this approach, stating that the assumptions are implausible and that it is not supported by neoclassical economic theory (S8, S9, S10). Also, even in developing countries with high unemployment rates, individuals outside the labor force can still be productive in informal employment or household sectors (S6). A recent review found that there was variation in the methods applied to estimate productivity cost across different countries (S11). Although both approaches have been criticized, the human capital approach is most often used (S12). Interestingly, the latest recommendations from the second US panel on cost-effectiveness in health and medicine suggested using the human capital approach (S6). They stated that this was because, from a societal perspective, the friction cost approach implies that the person who is compensating for the productivity losses of the sick individual was otherwise completely unproductive in the society, not only in terms of labor market production, but also in terms of the informal market and household production (S6).

Productivity costs can also result from lost unpaid work (services that are not officially sold in the formal market, such as household chores, caring for children and voluntary work). These are harder to capture and are often ignored (particularly for informal caregivers) (S2, S13). A complexity in this area is that it can be difficult to separate unpaid work from leisure time activities (for example caring for children may involve elements of both) (S2). A solution that is used to address this is to apply the ‘third person criterion’, where by all elements that are replaceable by a third person are considered unpaid work and all non-replaceable elements are considered leisure (S13).

There is continued debate as to whether lost leisure time should be valued within productivity costs (S14, S15).

The following papers provide further background information on productivity costs (S2, S3, S13, S14, S16, S17, S18, S19).

**References**

S1. Brouwer WB, Koopmanschap MA, Rutten FF. Productivity costs in cost‐effectiveness analysis: numerator or denominator: a further discussion. Health Econ. 1997;6(5):511-4.

S2. Krol M, Brouwer W, Rutten F. Productivity costs in economic evaluations: past, present, future. Pharmacoeconomics. 2013;31:537-49.

S3. Krol M, Brouwer W. How to estimate productivity costs in economic evaluations. Pharmacoeconomics. 2014;32:335-44.

S4. Gold MR, Siegel JE, Russell LB, Weinstein MC. Cost-effectiveness in health and medicine. New York: Oxford University Press; 1996.

S5. Goeree R, O'Brien BJ, Blackhouse G, Agro K, Goering P. The valuation of productivity costs due to premature mortality: a comparison of the human-capital and friction-cost methods for schizophrenia. The Canadian Journal of Psychiatry. 1999;44(5):455-63.

S6. Neumann PJ, Sanders GD, Russell LB, Siegel JE, Ganiats TG. Cost-effectiveness in health and medicine. Second ed: Oxford University Press; 2016.

S7. Koopmanschap MA, Rutten FF, van Ineveld BM, Van Roijen L. The friction cost method for measuring indirect costs of disease. J Health Econ. 1995;14(2):171-89.

S8. Van den Hout WB. The value of productivity: human-capital versus friction-cost method. Ann Rheum Dis. 2010;69(Suppl 1):i89-i91.

S9. Lensberg BR, Drummond, Danchenko, Despiegel N, Francois. Challenges in measuring and valuing productivity costs, and their relevance in mood disorders. ClinicoEconomics and Outcomes Research. 2013;5:565.

S10. Johannesson M, Karlsson G. The friction cost method: a comment. J Health Econ. 1997;16(2):249-55.

S11. Kigozi J, Jowett S, Lewis M, Barton P, Coast J. Estimating productivity costs using the friction cost approach in practice: a systematic review. Eur J Health Econ. 2016;17(1):31-44.

S12. Pritchard C, Sculpher MJ. Productivity costs: principles and practice in economic evaluation: Office of Health Economics London; 2000.

S13. Krol M, Brouwer W. Unpaid work in health economic evaluations. Soc Sci Med. 2015;144:127-37.

S14. Rothermich EA, Pathak DS. Productivity-cost controversies in cost-effectiveness analysis: review and research agenda. Clin Ther. 1999;21(1):255-67.

S15. Sendi P, Brouwer W. Leisure time in economic evaluation: theoretical and practical considerations. Expert Rev Pharmacoecon Outcomes Res. 2004;4(1):1-3.

S16. Jo C. Cost-of-illness studies: concepts, scopes, and methods. Clin Mol Hepatol. 2014;20:327-37.

S17. Brouwer WB, Koopmanschap MA, Rutten FF. Patient and informal caregiver time in cost-effectiveness analysis: a response to the recommendations of the Washington Panel. Int J Technol Assess Health Care. 1998;14(3):505-13.

S18. Olsen JA, Richardson J. Production gains from health care: what should be included in cost-effectiveness analyses? Soc Sci Med. 1999;49(1):17-26.

S19. Zhang W, Bansback N, Anis AH. Measuring and valuing productivity loss due to poor health: A critical review. Soc Sci Med. 2011;72:185-92.
