# Supplementary material for: Productivity costs from a dengue episode in Asia: a systematic literature review
Source: BMC Infect Dis. 2020 Jun 3;20:393. doi: 10.1186/s12879-020-05109-0 (PMC7268537; doi:10.1186/s12879-020-05109-0)
Supplement: Supplementary file 2 — Additional file 2 Description of the adjustment for inflation, Table S1: The estimated productivity losses of patients and their informal caregivers, Figure S1: The average reported productivity costs as a fraction of the study settings per capita GDP, The search terms and PRISMA CHECKLIST. [file 12879_2020_5109_MOESM2_ESM.docx]

**Supplementary Information: Productivity costs from a dengue episode in Asia: A systematic literature review**

**Overview of the adjustment for inflation**.

For the purpose of comparing the costs across studies, we adjusted the reported costs for inflation (to prices 2017). Generally, all of the reported costs were converted from US Dollars to the local currency using the exchange rate at the time the data were collected and then adjusted for inflation using the country’s Gross Domestic Product (GDP) deflators from the International Monetary Fund (S1). The costs were then converted back to US Dollars using the official exchange rate of 2017 from the World Bank (S2). It should be noted that owing to the differences in the exchange rates, the adjusted costs could also be lower than the original costs reported. For some specific cases It was possible to use the method outlined above:

Luh *et al.* (S3): For this study, it was not possible to adjust for inflation and we reported the original values.

Fezzazi *et al.* (S4): Fezzazi *et al.* (S4) did not report the productivity costs for every single country that they surveyed and reported the average US$ value across their study. We therefore used the United States GDP deflators to adjust for inflation.

Shepard *et al.* (S5) (Myanmar): Shepard et al. (s5) reported the productivity cost from several countries (including Myanmar). Because Myanmar had experienced rapid inflation during the relevant time period, we used the United States GDP deflators to adjust for inflation (without converting the cost reported to local currency).

| **Table S1: The estimated productivity losses of patients and their informal caregivers** | | | | |
| --- | --- | --- | --- | --- |
| **Authors** | **Asian countries** | **Target population and severity of disease** | **Productivity losses of patient (mean days)** **per dengue episode** | **Productivity losses of informal caregivers (mean days)** **per dengue** **episode** |
| **Hospitalized episodes** | | | | |
| Clark *et al.* (S6) | Thailand | Children and adults (DF) | Child and adult’s duration of illness: 9.1  Child’s school days lost: 4.8ᵡ  Adult: NA | Work days lost: 4.2 |
| Huy *et al.* (S7) | Cambodia | Children (Unclear) | Child’s days of illness: 8.1^ᵨ^ | Total days lost: 11.4  Work days lost: 8.3 |
| Mia *et al.* (S8) | Malaysia | Children and adults (DF) | Child and adult’s duration of illness: 9.7  Child’s school days lost: 6.25  Adult’s work days lost: 7.66 | Work days lost: 4.82 |
| Okanurak *et al.* ^µ^ (S9) | Thailand | Children and adults (DHF) | Child’s day of illness: 8  Adults: NA | Work days lost:  Main caregiver: 6  Assistant caregiver: 3.5 |
| Suaya *et al.* ^∏^ (S10) | Cambodia | Children  (DF and DHF) | Child’s school days lost: 6.8  Adult’s work days lost: NA | School days lost: 2.0  Work days lost: 14.3 |
|  | Thailand | Children  (DF and DHF) | Child’s school days lost: 5.5  Adult’s work days lost: NA | School days lost: 0  Work days lost: 3.9 |
|  | Malaysia | Children and adults (DF and DHF) | Child’s school days lost: 4.1  Adult’s work days lost: 8.8 | School days lost: 0.2  Work days lost: 2.9 |
| Kongsin *et al.* (S11) | Thailand | Children  (DF and DHF) | Child’s school days lost: 5.5 | Work days lost: 3.9 |
| Suaya *et al.* ^∏^ (S12) | Cambodia | Children  (DF and DHF) | Child’s school days lost: 6.6 | Work days lost: 14.2 |
| Pham *et al.* (S13) | Vietnam | Children and adults (DF) | Child and adults’ days lost: 7.8ᵡ | Days lost: 7.6 |
| Nguyen *et al.* (S14) | Vietnam | Children and adults  (DF and DHF) | Child: NA  Adults’ work days lost: 9.9 | Work days lost: 7.75 |
| Lee *et al.* (S15) | Vietnam | Children and adults  (DF and DHF) | Child and adult’s days lost:6.9 ^ᴪ^ | Days lost: 8.1 |
|  | Thailand | Children and adults  (DF and DHF) | Child and adult’s days lost: 6.1^ᴪ^ | Days lost: 2.1 |
| Rafique *et al.* (S16) | Pakistan | Adults  (DF and DHF) | Adult’s duration of illness: 31.9 | Days lost: 13 |
| Bhavsar *et al.* (S17) | India | Children  (DF and DHF) | Child’s school days lost: 11.4  Adults’ work days lost: 9.6 | Days lost of brothers/sister (<14 years old): 3.4  Adult’s days lost (>15 years old): 25.5 |
| Beaute *et al.* (S18) | Cambodia | Children and adults (unclear) | Children: NA  Adult’s work day loss: 5 | Work days lost: 5 |
| Tozan *et al.* (S19) | Thailand | Children and adults  (DF and DHF) | Child’s school days lost (<15 years old): 6.5  DF: 6.6  DHF: 6.1  Adult’s school days lost (>15 years old): 7.2  DF: 5.9  DHF: 10.3  Adult’s work days lost: 6.9  DF: 6.6  DHF: 7.6 | School days lost of household: 1.2  Adults’ work days lost: 4.1  Total mean days lost of the household: 7.2 |
| Fezzazi *et al.* (S4) | Indonesia | Children (DF) | Child’s school days lost: 5.3 | Work days lost: 1.7 |
|  | Malaysia | Children (DF) | Child’s school days lost: 5.2 | Work days lost: 1.4 |
|  | Philippines | Children (DF) | Child’s school days lost: 5.6 | Work days lost: 1.0 |
|  | Thailand | Children (DF) | Child’s school days lost: 3.8 | Work days lost: 3.3 |
|  | Vietnam | Children (DF) | Child’s school days lost: 3.9 | Work days lost: 2.9 |
| Bach *et al.* (S20) | Vietnam | Adults (DF) | Adult’s days of illness: 5.9 | NA |
| Anderson *et al.* (S21) | Thailand | Children  (DF and DHF) | Child’s duration of illness:  DHF: 8.4  DF: 6.3 | NA |
| Lee *et al.* (S22) | Cambodia | Children and adults (Unclear) | Child and adults’ days of illness:  Child: 8.6  Adult: 9.1 | NA |
| **Outpatient episodes** | | | | |
| Suaya *et al.* ^∏^ (S10) | Malaysia | Children and adults  (DF and DHF) | Number of illness days: 8.6  Child’s school days lost: 3.2  Adult’s work days lost: 7.2. | School days lost: 0.3  Work days lost: 1.6 |
| Lee *et al.* (S15) | Vietnam | Children and adults  (DF and DHF) | Child and adult’s day loss: 5.7 ^ᴪ^ | Days lost: 2.0 |
|  | Thailand | Children and adults  (DF and DHF) | Child and adult’s day loss: 4.0 ^ᴪ^ | Days lost: 2.0 |
| Fezzazi *et al.* (S4) | Indonesia | Children (DF) | Child’s school days lost: 2.1 | Work days lost: 0.1 |
|  | Malaysia | Children (DF) | Child’s school days lost: 1.4 | Work days lost: 0.3 |
|  | Philippines | Children (DF) | Child’s school days lost: 1.9 | Work days lost: 0.3 |
|  | Thailand | Children (DF) | Child’s school days lost: 2.6 | Work days lost: 1.9 |
|  | Vietnam | Children (DF) | Child’s school days lost: 0.7 | Work days lost: 0.6 |
| Bach *et al.* (S20) | Vietnam | Adults (DF) | Adult’s days of illness: 4.9 | NA |
| Anderson *et al.* (S21) | Thailand | Children  (DF and DHF) | Child’s duration of illness: 4.3 | NA |
| **Unspecified setting** | | | | |
| Luh *et al.* (S3) | Taiwan | Children and adults (DF and DHF) | Child (Caregiver’s work days lost):  DF: 2.2  DHF: 3.7  Adult’s work days lost:  DF: 5.4  DHF: 9.8 | NA |
| Carrasco *et al.* (S23) | Singapore | Children and adults (DF and DHF) | Child and adults’ duration of illness:  DF: 10  DHF: 14 | NA |
| *^ᵨ^: Days were accounted for ferbile illness of dengue and non-dengue cases.*  *ᵡ: The average number of school days lost or work days lost by the patients.*  *NA: Not available. DF: Dengue Fever; DHF: Dengue Hemorrhagic Fever.*  *^µ^: Because there were less than 3% of patients were older than 14, we assumed that the number of illness days reported were days lost of children.*  *^∏^: The work day loss of caregiver was accounted for the household.*  *^ᴪ:^ The average of number of days lost due to illness for all patients older than 5.* | | | | |


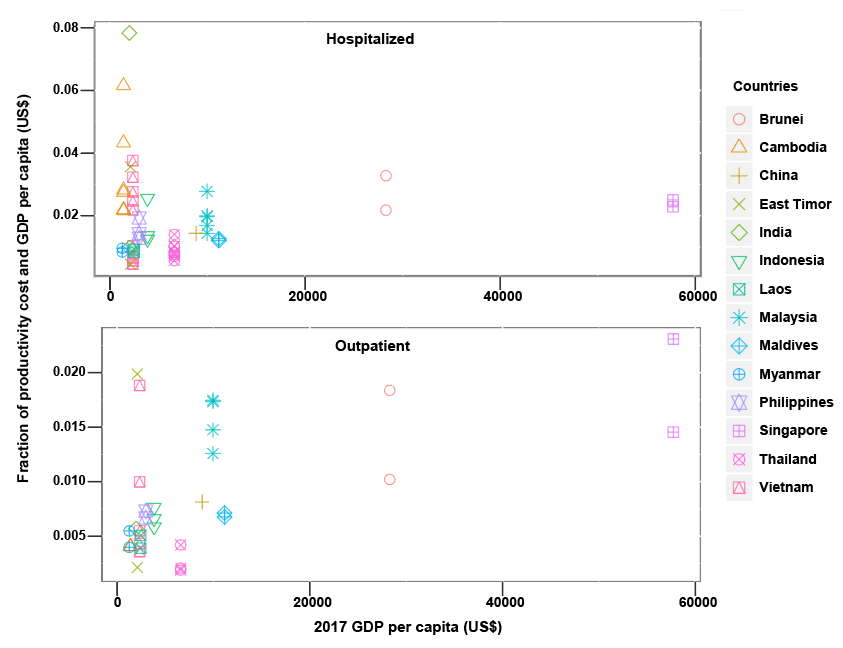


**Figure S1**: **The average reported productivity costs as a fraction of the study settings per capita GDP.** *Costs are reported in 2017 prices.*

**Search term**

("dengue"[MeSH Terms] OR "dengue"[All Fields] OR "dengue hemorrhagic fever"[All Fields] OR "dengue fever"[All Fields] OR "dengue infection"[All Fields] OR "dengue infection"[All Fields] OR "dengue infections"[All Fields] OR ("dengue virus"[MeSH Terms] OR ("dengue"[All Fields] AND "virus"[All Fields]) OR "dengue virus"[All Fields]) OR (DENV[All Fields] AND ("viruses"[MeSH Terms] OR "viruses"[All Fields] OR "virus"[All Fields])) OR DENV-1[All Fields] OR "dengue 1 virus"[All Fields] OR "dengue virus type 1"[All Fields] OR DENV-2[All Fields] OR "dengue 2 virus"[All Fields] OR "dengue virus type 2"[All Fields] OR DENV-3[All Fields] OR "dengue 3 virus"[All Fields] OR "dengue virus type 3"[All Fields] OR DENV-3[All Fields] OR "dengue 3 virus"[All Fields] OR "dengue virus type 3"[All Fields] OR DENV-4[All Fields] OR "dengue 4 virus"[All Fields] OR "dengue virus type 4"[All Fields] OR DENV-4[All Fields] OR "dengue 4 virus"[All Fields] OR "dengue virus type 4"[All Fields]) AND (("economics"[Subheading] OR "economics"[All Fields] OR "cost"[All Fields] OR "costs and cost analysis"[MeSH Terms] OR ("costs"[All Fields] AND "cost"[All Fields] AND "analysis"[All Fields]) OR "costs and cost analysis"[All Fields] OR "cost analysis"[All Fields]) OR ("health care costs"[MeSH Terms] OR ("health"[All Fields] AND "care"[All Fields] AND "costs"[All Fields]) OR "health care costs"[All Fields] OR ("health"[All Fields] AND "care"[All Fields] AND "cost"[All Fields]) OR "health care cost"[All Fields]) OR ("absenteeism"[MeSH Terms] OR "absenteeism"[All Fields]) OR ("presenteeism"[MeSH Terms] OR "presenteeism"[All Fields]) OR ("cost of illness"[MeSH Terms] OR ("cost"[All Fields] AND "illness"[All Fields]) OR "cost of illness"[All Fields] OR ("disease"[All Fields] AND "cost"[All Fields]) OR "disease cost"[All Fields]) OR "Indirect cost"[All Fields] OR ("cost-benefit analysis"[MeSH Terms] OR ("cost-benefit"[All Fields] AND "analysis"[All Fields]) OR "cost-benefit analysis"[All Fields] OR ("cost"[All Fields] AND "benefit"[All Fields]) OR "cost benefit"[All Fields]) OR (("cost"[All Fields] AND "effectiveness"[All Fields]) OR "cost effectiveness"[All Fields]))

**PRISMA CHECKLIST**

| **Section/topic** | **#** | **Checklist item** | **Reported on page #** |
| --- | --- | --- | --- |
| **TITLE** | | |  |
| Title | 1 | Identify the report as a systematic review, meta-analysis, or both. | 1 |
| **ABSTRACT** | | |  |
| Structured summary | 2 | Provide a structured summary including, as applicable: background; objectives; data sources; study eligibility criteria, participants, and interventions; study appraisal and synthesis methods; results; limitations; conclusions and implications of key findings; systematic review registration number. | 2 |
| **INTRODUCTION** | | |  |
| Rationale | 3 | Describe the rationale for the review in the context of what is already known. | 3 |
| Objectives | 4 | Provide an explicit statement of questions being addressed with reference to participants, interventions, comparisons, outcomes, and study design (PICOS). | NA |
| **METHODS** | | |  |
| Protocol and registration | 5 | Indicate if a review protocol exists, if and where it can be accessed (e.g., Web address), and, if available, provide registration information including registration number. | NA |
| Eligibility criteria | 6 | Specify study characteristics (e.g., PICOS, length of follow-up) and report characteristics (e.g., years considered, language, publication status) used as criteria for eligibility, giving rationale. | 4 |
| Information sources | 7 | Describe all information sources (e.g., databases with dates of coverage, contact with study authors to identify additional studies) in the search and date last searched. | 4 |
| Search | 8 | Present full electronic search strategy for at least one database, including any limits used, such that it could be repeated. | 4 |
| Study selection | 9 | State the process for selecting studies (i.e., screening, eligibility, included in systematic review, and, if applicable, included in the meta-analysis). | 4 |
| Data collection process | 10 | Describe method of data extraction from reports (e.g., piloted forms, independently, in duplicate) and any processes for obtaining and confirming data from investigators. | 4 |
| Data items | 11 | List and define all variables for which data were sought (e.g., PICOS, funding sources) and any assumptions and simplifications made. | NA |
| Risk of bias in individual studies | 12 | Describe methods used for assessing risk of bias of individual studies (including specification of whether this was done at the study or outcome level), and how this information is to be used in any data synthesis. | NA |
| Summary measures | 13 | State the principal summary measures (e.g., risk ratio, difference in means). | NA |
| Synthesis of results | 14 | Describe the methods of handling data and combining results of studies, if done, including measures of consistency (e.g., I^2^) for each meta-analysis. | NA |

**References**

S1. International Monetary Fund. Gross Domestic Product, Deflator 2018 [Available from: <https://www.imf.org/external/pubs/ft/weo/2018/02/weodata/weorept.aspx?sy=1990&ey=2023&scsm=1&ssd=1&sort=country&ds=.&br=1&c=512%2C548%2C556%2C513%2C514%2C518%2C516%2C558%2C522%2C564%2C924%2C566%2C576%2C528%2C532%2C578%2C534%2C537%2C536%2C429%2C433%2C158%2C542%2C927%2C544%2C582&s=NGDP_D&grp=0&a=&pr1.x=69&pr1.y=6>.

S2. World Bank. Official exchange rate 2018 [Available from: <https://data.worldbank.org/indicator/PA.NUS.FCRF?end=2017&locations=AF-BD-BT-BN-CN-KH-IN-ID-IR-JP-KR-LA-MY-MV-MM-NP-PK-PH-SG-UZ-VN&start=2013>.

S3. Luh DL, Liu CC, Luo YR, Chen SC. Economic cost and burden of dengue during epidemics and non-epidemic years in Taiwan. J Infect Public Health. 2017.

S4. El Fezzazi H, Branchu M, Carrasquilla G, Pitisuttithum P, Perroud AP, Frago C, et al. Resource Use and Costs of Dengue: Analysis of Data from Phase III Efficacy Studies of a Tetravalent Dengue Vaccine. Am J Trop Med Hyg. 2017;97(6):1898-903.

S5. Shepard DS, Undurraga EA, Halasa YA. Economic and Disease Burden of Dengue in Southeast Asia. PLoS Negl Trop Dis. 2013;7:e2055.

S6. Clark DV, Mammen MP, Jr., Nisalak A, Puthimethee V, Endy TP. Economic impact of dengue fever/dengue hemorrhagic fever in Thailand at the family and population levels. Am J Trop Med Hyg. 2005;72(6):786-91.

S7. Huy R, Wichmann O, Beatty M, Ngan C, Duong S, Margolis HS, et al. Cost of dengue and other febrile illnesses to households in rural Cambodia: a prospective community-based case-control study. BMC Public Health. 2009;9:155.

S8. Mia MS, Begum RA, Er AC, Pereira JJ. Assessing the cost burden of dengue infection to households in Seremban, Malaysia. Southeast Asian J Trop Med Public Health. 2016;47(6):1167-76.

S9. Okanurak K, Sornmani S, Indaratna K. The cost of dengue hemorrhagic fever in Thailand. Southeast Asian J Trop Med Public Health. 1997;28(4):711-7.

S10. Suaya JA, Shepard DS, Siqueira JB, Martelli CT, Lum LC, Tan LH, et al. Cost of dengue cases in eight countries in the Americas and Asia: a prospective study. Am J Trop Med Hyg. 2009;80(5):846-55.

S11. Kongsin S, Jiamton S, Suaya JA, Vasanawathana S, Sirisuvan P, Shepard DS. Cost of dengue in Thailand. Dengue Bull. 2010;34:77-88.

S12. Suaya JA, Chantha N, Huy R, Sah BK, Moh-Seng C, Socheat D, et al. Clinical characterization, diagnosis and socioeconomic impact of hospitalized dengue in Cambodia. Dengue Bull. 2010;34:89-102.

S13. Pham LD, Tran NHP, Le NDT, Vo TQ. Economic report on the cost of dengue fever in Vietnam: case of a provincial hospital. Clinicoecon Outcomes Res. 2016;9:1-8.

S14. Nguyen TKT, Luong CQ. Assessing the economic burden of dengue in Southern Viet Nam: Results of a prospective multicenter cost study. 2011.

S15. Lee JS, Mogasale V, Lim JK, Carabali M, Lee KS, Sirivichayakul C, et al. A multi-country study of the economic burden of dengue fever: Vietnam, Thailand, and Colombia. PLoS Negl Trop Dis. 2017;11(10):e0006037.

S16. Rafique I, Nadeem Saqib MA, Munir MA, Qureshi H, Siddiqui S, Habibullah S, et al. Economic burden of dengue in four major cities of Pakistan during 2011. J Pak Med Assoc. 2015;65(3):256-9.

S17. Bhavsar AT, Shepard DS, Suaya JA, Mafowosofo M, Hurley CL. A private hospital-based study assessing knowledge, attitudes, practices and costs associated with dengue illness in Surat, India. Dengue Bull. 2010;34:54-64.

S18. Beaute J, Vong S. Cost and disease burden of dengue in Cambodia. BMC Public Health. 2010;10:521.

S19. Tozan Y, Ratanawong P, Sewe MO, Wilder-Smith A, Kittayapong P. Household costs of hospitalized dengue illness in semi-rural Thailand. PLoS Negl Trop Dis. 2017;11(9):e0005961.

S20. Tran BX, Thu GV, Hoang LN, Tuan ALN, Thanh TT, Thanh BN, et al. Cost-of-Illness and the Health-Related Quality of Life of Patients in the Dengue Fever Outbreak in Hanoi in 2017. Int J Environ Res Public Health. 2018;15(6).

S21. Anderson KB, Chunsuttiwat S, Nisalak A, Mammen MP, Libraty DH, Rothman AL, et al. Burden of symptomatic dengue infection in children at primary school in Thailand: a prospective study. The Lancet. 2007;369(9571):1452-9.

S22. Lee J-S, Mogasale V, Lim JK, Ly S, Lee KS, Sorn S, et al. A multi-country study of the economic burden of dengue fever based on patient-specific field surveys in Burkina Faso, Kenya, and Cambodia. PLoS Negl Trop Dis. 2019;13(2):e0007164.

S23. Carrasco LR, Lee LK, Lee VJ, Ooi EE, Shepard DS, Thein TL, et al. Economic impact of dengue illness and the cost-effectiveness of future vaccination programs in Singapore. PLoS Negl Trop Dis. 2011;5(12):e1426.
